# Response of Southeast Asian rice root architecture and anatomy phenotypes to drought stress

**DOI:** 10.3389/fpls.2022.1008954

**Published:** 2022-10-19

**Authors:** Jonaliza L. Siangliw, Burin Thunnom, Mignon A. Natividad, Marinell R. Quintana, Dmytro Chebotarov, Kenneth L. McNally, Jonathan P. Lynch, Kathleen M. Brown, Amelia Henry

**Affiliations:** ^1^ National Center for Genetic Engineering and Biotechnology, National Science and Technology Development Agency, Pathum Thani, Thailand; ^2^ Rice Breeding Innovations Platform, International Rice Research Institute, Los Baños, Philippines; ^3^ Department of Plant Science, The Pennsylvania State University, University Park, PA, United States

**Keywords:** GWAS, drought, rice, root anatomy, root architecture

## Abstract

Drought stress in Southeast Asia greatly affects rice production, and the rice root system plays a substantial role in avoiding drought stress. In this study, we examined the phenotypic and genetic correlations among root anatomical, morphological, and agronomic phenotypes over multiple field seasons. A set of >200 rice accessions from Southeast Asia (a subset of the 3000 Rice Genomes Project) was characterized with the aim to identify root morphological and anatomical phenotypes related to productivity under drought stress. Drought stress resulted in slight increases in the basal metaxylem and stele diameter of nodal roots. Although few direct correlations between root phenotypes and grain yield were identified, biomass was consistently positively correlated with crown root number and negatively correlated with stele diameter. The accessions with highest grain yield were characterized by higher crown root numbers and median metaxylem diameter and smaller stele diameter. Genome-wide association study (GWAS) revealed 162 and 210 significant SNPs associated with root phenotypes in the two seasons which resulted in identification of 59 candidate genes related to root development. The gene *OsRSL3* was found in a QTL region for median metaxylem diameter. Four SNPs in *OsRSL3* were found that caused amino acid changes and significantly associated with the root phenotype. Based on the haplotype analysis for median metaxylem diameter, the rice accessions studied were classified into five allele combinations in order to identify the most favorable haplotypes. The candidate genes and favorable haplotypes provide information useful for the genetic improvement of root phenotypes under drought stress.

## 1 Introduction

As the major rice production area in the world, Southeast Asia relies on agriculture as the primary source of income, and climate change will have a significant impact on agricultural production. Since many rice planting areas in Southeast Asia rely on rain, drought is considered as a major problem in rice production in Southeast Asia. In the case of Northeast Thailand, rainfed lowland rice cultivation covers approximately 75% of the agricultural area in Northeast Thailand (Jongdee et al., 2006), and drought stress constraining rice production in that region is due to unpredictable seasonal rains (Jongdee, 2001). Yield loss due to drought in Thailand is estimated at 55-68% (Polthanee and Promkhambut, 2014), and the continuous decline in rainfall in the country from 2010 to 2016 (Thaiturapaisan, 2016) indicates that even greater reductions in future rice production are to be expected unless new rice varieties are developed that can produce more grain yield with less water.

To develop drought tolerant rice varieties suitable for cultivation in Southeast Asia, key drought tolerance phenotypes and their related genetic regions should be identified. As the most significant tissues involved in moisture and nutrient absorption, the properties, structure, and distribution of roots are interesting to study in the context of improving drought tolerance. Root architectural phenotypes function in penetrating, exploring, and absorbing water and nutrients from the soil which in drought-prone areas tends to be most beneficial for water in the deep soil layers (Lynch, 2015; Lynch and Wojciechowski, 2015). Some examples of root architectural phenotypes related to yield under drought in rice include greater root length density at depth (as reviewed by Gowda et al., 2011), and deeper root angle as conferred by an allele of the QTL *DRO1* Uga et al. (2013). The relationship between root crown phenotypes and drought response in rice, however, appear to be less clear-cut: lower crown root numbers and proportion or shallow root growth under drought stress were observed to be conferred by the major-effect drought-yield QTL *qDTY_3.2_* (Grondin et al., 2018), but an increase in the total number of nodal roots resulted in increased biomass under drought in a set of upland and lowland varieties from Myanmar (Sandar et al., 2022).

Root anatomical phenotypes such as diameter, cortical area, and size of xylem vessels influence the metabolic cost of soil exploration and the rate of water and nutrient transport in plants (Schneider et al., 2017; Lynch et al., 2021). The xylem vessels also influence root hydraulic conductivity and therefore are associated with water use efficiency (Rieger and Litvin, 1999). Genetic variation and the benefits associated with root anatomy have been observed in wheat (Richards and Passioura, 1989), maize (Lynch et al., 2014; Saengwilai et al., 2014), bean (Strock et al., 2020), and rice (Uga et al., 2008; Uga et al., 2009; Singh et al., 2013), with a focus on nodal (axial) roots. Increase in root diameter was found to be associated with drought avoidance and resulted in better penetration ability in deeper soil layers (as reviewed by Comas et al., 2013). Kadam et al. (2015) reported that wheat produced larger diameter roots than rice, and that the anatomy of wheat roots was more responsive to drought than that of rice. However, the location of root anatomical measurements along the root axis (i.e. basal vs. apical) can have a strong influence on the responses observed. In measurements at the mid-point of the root axis under drought, Henry et al. (2012) reported greater rice stele diameter when the soil moisture was reduced, and this finding was complemented by the study of Phoura et al. (2020) that observed basal stele transversal area to be increased in IR64 lines introgressed with Stele Traversal Area 1 (Sta 1) under drought stress. On the other hand, Richards and Passioura (1989) reported smaller basal xylem vessels (indicating smaller stele size) reduced water use of wheat under drought resulting in saving more water during grain filling to attain higher grain yield. Smaller and fewer xylem vessels were observed near the rice root apex under drought by Henry et al. (2012), but other studies observed mixed responses depending on the experiment and the accessions studied (Fonta et al., 2022a).

The objectives of this study were to identify favorable phenotypes and haplotypes that could be used in breeding for improved rice productivity in drought-prone rice-growing regions with a focus on Southeast Asia. We characterized the drought response and root phenotypes (with an emphasis on anatomical phenotypes measured in basal nodal root segments), and their associated genetic regions, in a subset of the 3000 Rice Genomes (The 3000 Rice Genomes Project, 2014) originating from Southeast Asia. Experiments were conducted under both puddled transplanted and direct seeded conditions, both of which are establishment methods used by farmers in drought-prone areas of Southeast Asia. Our aim with selecting this set of accessions was to increase the likelihood that use of identified lines with favorable root phenotypes in breeding would generate cultivars that could more readily be adapted to the environmental conditions in drought-prone rice-growing regions of Thailand.

## 2 Materials and methods

### 2.1 Accessions studied

A set of >200 accessions from the 3000 Rice Genomes Project (The 3000 Rice Genomes Project, 2014) were selected for this study based on their passport data indicating origin in Southeast Asia [Cambodia, Lao PDR, Myanmar, Thailand and Vietnam (**Table S1**)], and for having the maximum available sequencing depth relative to Nipponbare. The full subset was grown in field trials under puddled, transplanted conditions in the 2016 wet season (WS) and under dry-direct seeded conditions in the 2018WS. In the 2017 dry season (DS), 2017WS, and 2018DS, puddled, transplanted field trials were grown using smaller subsets of accessions chosen for their similar phenology and contrasting drought response (**Table S1**). KDML 105 (the most popular variety in Thailand mainly grown in the drought-prone Northeast region; Vanavichit et al., 2018) was included as a check in all experiments, and drought-tolerant Sew mae chan (or “Sew mae jan”; Somrith and Prommani, 1986) was also included as a check in the 2018WS experiment.

### 2.2 Field experiments

A series of field experiments were conducted at the Zeigler Experimental Station of the International Rice Research Institute (IRRI; 14°10’11.81” N, 121°15’39.22” E) to investigate root traits related to performance under drought (**Table 1**). For the experiments in which the full subset of accessions was planted, an augmented experimental design was used in 2016WS with two 3-m rows per plot, and an alpha lattice design was used in 2018WS with three 3-m rows per plot. For the experiments with smaller subsets, four 3-m rows were planted in 2017DS and 2018DS, and three 3-m rows per plot were planted in a randomized complete block design in 2017WS. The puddled, transplanted experiments were sown in a seed bed and transplanted to the main experimental field at about 17 days after sowing and irrigated by flooding. The dry-direct seeded experiment was sown directly into harrowed, dry soil and irrigated by sprinkler. All well-watered treatments were irrigated 2-3 times per week throughout the season. All drought stress treatments were planted in automated rainout shelters. The drought stress treatments were initiated at 38 days after sowing (das) in both experiments using the full subset of accessions (2016WS and 2018WS), and at 40, 53, and 60 days in the experiments on the early- (2017DS), medium- (2017WS), and late- (2018DS) maturing smaller subsets. The drought treatments were rewatered 1-2 times per season to ensure survival of as many accessions as possible. Soil moisture levels were recorded in all experiments by tensiometers (1-2 per replicate/block; Soilmoisture Equipment Co.) installed at a soil depth of 30 cm and by frequency domain reflectometry (one per replicate; Diviner 2000, Sentek Sensor Technologies) through PVC tubes installed to a depth of 70 cm.

**Table 1:** List of experiments and measurements conducted in this study.

Season	# Accessions	Expt design (# replicates)	Root measurements conducted
2016WS	211	Augmented (1)	Root crowns: Crown root number, # crown roots per tiller, stele diameter, median metaxylem diameter, number of metaxylem, plasticity in stele diameter, median metaxylem diameter, number of metaxylem
2017DS	18 (early maturing)	RCBD (3)	Root crowns: Crown root number, # crown roots per tiller, stele diameter, median metaxylem diameter, number of metaxylem, plasticity in stele diameter, median metaxylem diameter, number of metaxylem Soil cores: shallow (0-0 cm depth) and deep (30-60 cm depth) S-type lateral, L-type lateral, and nodal root length density
2017WS	18 (medium duration)	RCBD (3)	
2018DS	9 (late maturing)	RCBD (4)	
2018WS	223	Alpha lattice (3)	Root crowns: Crown root number, # crown roots per tiller, stele diameter, median metaxylem diameter, number of metaxylem, plasticity in stele diameter, median metaxylem diameter, number of metaxylem Soil cores: shallow (0-30 cm depth) and deep (30-60 cm depth) S-type lateral, L-type lateral, and nodal root length density

All experiments included a drought and a well-watered treatment. WS, wet season; DS, dry season; RCBD, randomized complete block design.

### 2.3 Phenotypic characterization

#### 2.3.1 Root phenotypes

Root crowns (one hill per plot in transplanted experiments and one plant per plot in the 2018 direct-seeded experiment) were sampled at 48-52 das in the experiments using the full subset of accessions (2016WS and 2018WS), and at 76-82 das in the experiments on the smaller subsets (2017DS-2018DS). The root crowns were manually sampled with a spade to a depth of about 25 cm and a distance of 25 cm from the base of the plant, then gently washed with tap water. The number of crown (nodal) roots and the number of tillers were counted from each root crown. Each root crown from the 2016WS-2018DS experiments was then imaged together with a size standard and analyzed in DIRT (Bucksch et al., 2014) to determine the root crown top angle and density. The increase in top angle under drought was calculated as top angle_drought stress_ – top angle_well-watered_.

Soil cores (4-cm diameter, 60 cm length; Giddings, USA) were sampled half-way between rows from three locations per plot in the experiments on the smaller subsets at 77-82 das in the 2017DS, 2017WS, and 2018DS, and from one location per plot in the 2018WS experiment at 56 das using the full subset of accessions. Each soil core was divided into 15-cm depth segments, and roots were gently washed from the soil and scanned at 600 dpi (WinRhizo Pro v. 2013e) to determine root length and root length within diameter classes. We considered S-type lateral roots as those with a diameter of <0.05 mm, L-type lateral roots as those with a diameter of 0.05-0.2 mm, and nodal roots as those with a diameter >0.2 mm. The “percent deep roots” was calculated as the total length in the soil core below a depth of 30 cm divided by the total root length at all depths in the soil core, multiplied by 100. Percent deep root increase under drought was calculated as percent deep roots_drought stress_ – percent deep roots_well-watered_.

Root anatomical parameters were measured in all experiments using basal nodal root segments (about 1-4 cm from the root-shoot junction) from the root crown samples which were stored in 75% ethanol. Based on the infolding of the cortex in root samples from 2016WS, the nodal root segments in 2017DS, 2017WS, and 2018DS were prepared by critical point drying using an ethanol series incubation to remove the water. Since that approach did not appear to resolve the infolding, the root anatomy was again analyzed on roots stored in ethanol in 2018WS.

For the critical point drying, samples were incubated for one hour in 80%, 90%, and 100% ethanol, respectively. At 30 minutes before the one-hour incubation in 100% ethanol was completed, preliminary operation of the critical point dryer was performed to hasten the temperature drop. The temperature was set to -10°C, and then the chamber was filled with 50-80% CO_2_ until the pressure reached 50-70 kg cm^-2^. Inlet valves were closed, and the exhaust was opened until the pressure dropped to zero. This applying and releasing of pressure was done four times. Root samples were cut into 2-cm length segments, placed in a histocap and put into the chamber with a few drops of 100% ethanol. The chamber cap and all valves were closed. Temperature was set to -10°C, and then substitution with CO_2_ was performed by applying pressure at 50-70 kg cm^-2^ and gently opening the exhaust until no moisture was detected. The exhaust valve was closed, and pressure was applied again allowing the samples to sit for 30 minutes. Evaporation was done by setting the temperature to 37°C until the pressure increased up to 100 kg cm^-2^. Leak valves were opened to set the discharge rate. Before removing the samples, the temperature was set to room temperature.

Samples were sent to PennState where basal root segments were sectioned, imaged, and analyzed with laser ablation tomography (LAT) as described in Strock et al. (2019) and Hall et al. (2019). Stele anatomical parameters including stele diameter, metaxylem number, and metaxylem diameter were analyzed on all root samples. The cortex and epidermis were not measured due to distortion of the samples that is typical of soil-grown rice roots (see Hazman and Brown, 2018). All root cross-section images were analyzed using Image J (version 1.45s).

#### 2.3.2 Agronomic phenotypes

At maturity, an area of 0.8 m^2^ (16 hills) in 2016WS, 1.5 m^2^ (30 hills) in 2017DS-2018DS, and 1 m^2^ in 2018WS was harvested from each plot. Straw biomass and grain yield (normalized to a 14% moisture content) were determined from each plot. Harvest index was calculated as grain yield/(grain yield + straw biomass).

#### 2.3.3 Phenotypic analysis and PCA regression, AMMI, path coefficient analysis

Root anatomical phenotype reduction was calculated on the lsmeans for each accession as $\frac{\;\;{\bar{x}}_{well-watered}-{\bar{x}}_{drought\;stress}}{{\bar{x}}_{well-watered}}$ . In the experiments on the smaller set of accessions, L-type lateral root plasticity was calculated as $\frac{\;x_{drought\;stress}-\;{\bar{x}}_{well-watered}}{{\bar{x}}_{well-watered}}$ .

To identify correlated phenotypes across the three experiments on smaller subsets based on maturity group (2017WS-2018DS), principal component regression was conducted according to Jolliffe (2002). First, we calculated lsmeans for each accession/phenotype/experiment. Using the lsmeans, we ran a principal component analysis, followed by a multiple regression on the principal components in order to identify phenotypes with the largest loading values from each of the PCA rotations.

Broad-sense heritability (H^2^) was determined on phenotypes measured in 2016WS and 2018WS using PBTools 1.4 (bbi.irri.org). To determine the most stable and highest-yielding accessions in terms of grain yield, an additive main effects and multiplicative interaction (AMMI) analysis (Annicchiarico, 1997; Zhang et al., 1998) was conducted across the drought and well-watered treatments from the 2016WS and 2018WS experiments (i.e. four environments). The AMMI analysis was conducted using Plant Breeding Tools (PB Tools) v. 1.3 (bbi.irri.org). The AMMI model used was:


$$P_{ij}=\mu +\;{\text{τ}}_{\text{i}}+{\text{δ}}_{\text{j}}+\sum_{\text{k}=1}^{\text{t}}{\text{λ}}_{\text{k}}{\text{α}}_{\text{ij}}{\text{γ}}_{\text{ij}}{\text{ϵ}}_{\text{ij}}$$

P_ij_ is the grain yield, µ is the grand mean; τ_i_ is the genotypic effect; δ_j_ is the environmental effect; the constant λ_k_ is the singular value of the k^th^ bilinear (multiplicative) component that is ordered λ_1_≥λ_2_ ≥…≥λ_t_; α_ik_ are elements of the k^th^ left singular vector of the true interaction and represent genotypic sensitivity to hypothetical environmental factors represented by the k^th^ right singular vector with elements γ_jk_. The terms α_ik_ and γ_jk_ satisfy the constraints $\sum_{i=1}^{g}{\text{α}}_{ik}{\text{α}}_{{\text{ik}}^{"}}=\sum_{j=1}^{s}{\text{γ}}_{jk}{\text{γ}}_{{\text{jk}}^{"}}=0$ for k≠k*’* and $\sum_{i}{\text{α}}_{ik}^{2}=\sum_{j}{\text{γ}}_{jk}^{2}=1$ .

To examine inter-related phenotypes in the experiments using the full subset of accessions (2016WS and 2018WS), path coefficient and Pearson’s r correlation were analyzed by GenStat 19th Edition software (Payne, 2009) for all treatments.

#### 2.3.4 GWAS: Genotype data preparation

The genotype data were downloaded from the 3K rice genomes 1M GWAS SNP dataset available at https://snp-seek.irri.org. A total of 1,011,601 SNPs were identified for the root phenotypes and agronomic phenotypes of the 210 rice accessions. Missing data of 10% or more were filtered out using PLINK. The resulting genotype data were further filtered using 5% minor allele frequency as the criterion. In the end, there were 462,202 SNPs that were used in determining the population structure that were estimated by PCA using the software TASSEL (Bradbury et al., 2007). The PCA revealed a strong population structure separating the indica rice from the japonica rice accessions. The indica group was comprised of 164 varieties that were used in identifying QTL using GWAS with 369,070 SNP markers determined for this group. Association analysis of the 164 indica accessions was performed with TASSEL using Mixed Linear Model (MLM) methods. Marker alleles in the MLM model were declared to be significantly associated with the phenotypes using two significance thresholds - a stricter value (-log10(p) = 5) in searching for candidate genes specific to the phenotype, and a less strict value (-log10(p) =4), for colocations.

#### 2.3.5 GO ontology enrichment and gene colocation

Gene ontology (GO) enrichment analysis was carried out to further characterize the main molecular functions, biological processes, cellular components and protein classes of gene sets from each QTL region. GO enrichment analysis was performed using http://geneontology.org/ by entering the assigned gene LOC codes obtained after gene annotation. Significance values for the GO terms were determined in PlantGSEA (Yi et al., 2013).

#### 2.3.6 Haplotype analysis and candidate genes

From the GWAS results, linkage disequilibrium blocks and haplotypes of significantly associated SNPs were examined. Haploview software was implemented with the solid spline method for defining blocks in each chromosome (Barrett et al., 2005). After blocks were identified, we used R version 4.0.3 to conduct a multiple linear regression (He and Zelikovsky, 2006) of SNPs, including the GWAS SNP and other SNPs within the block to investigate any effect or association of other SNPs within the block with the phenotype. Multiple iterations were performed until the significant SNPs were not changed. In case there were more than one SNP remaining in a block, the tree-based SNP-SNP interaction analysis (Li et al., 2015) was subsequently performed. Moreover, if there were several blocks with significant SNPs in a phenotype, the SNPs were combined, and multiple regression was again performed followed by n-way interaction analysis of the remaining significant SNPs.

## 3 Results

### 3.1 Drought response of agronomic and root phenotypes

The drought stress progressed over time in all puddled, transplanted experiments as evidenced by tensiometer and volumetric soil moisture measurements at a depth of 30 cm (**Figure S1**). In comparison, the decline in soil moisture at 30 cm was not as evident in the 2018WS direct-seeded drought experiment. In the experiments on the full set of accessions, the agronomic phenotypes (biomass, grain yield and harvest index) were all reduced by drought stress in all experiments (**Figure S2**). The highest biomass under well-watered conditions was 1540 g m^-2^ in 2016 while the maximum biomass obtained under stress was 783 g m^-2^, and the grain yield was reduced to half in the drought stress treatment. In 2018, the biomass was reduced by more than half under drought stress, with the highest biomass amounting to 871 g m^-2^ (**Figure S2**). The low biomass values despite relatively high soil moisture readings in the 2018WS drought treatment are likely due the continuous aerobic conditions of the soil, which causes growth reduction and is common in direct-seeded, aerobic rice (Nie et al., 2008). Harvest index in both years followed the same trend as grain yield. The drought stress treatments also had a consistent effect on agronomic phenotypes in the experiments on smaller subsets, with average grain yield reduced by 87-90% compared to the well-watered treatment (**Table S2**). Among agronomic traits, H^2^ was highest for biomass, ranging from 0.31-0.91 (**Figure S2**).

**Figure 1:**
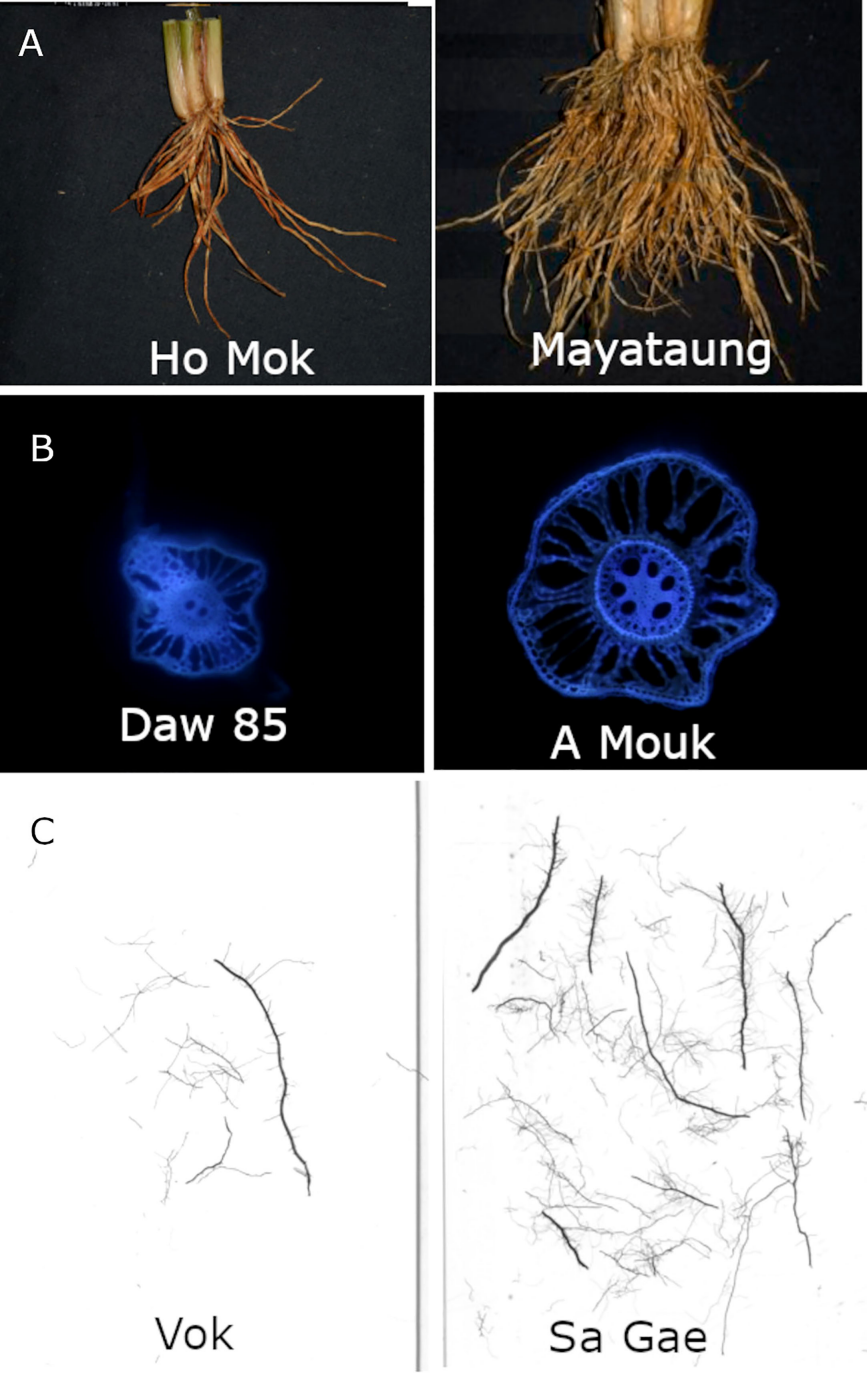
Example accessions with contrasting values for key phenotypes in this study: **(A)** crown root number, **(B)** stele diameter, **(C)** shallow S-type lateral root length. Accession names are shown in each image.

**Figure 2:**
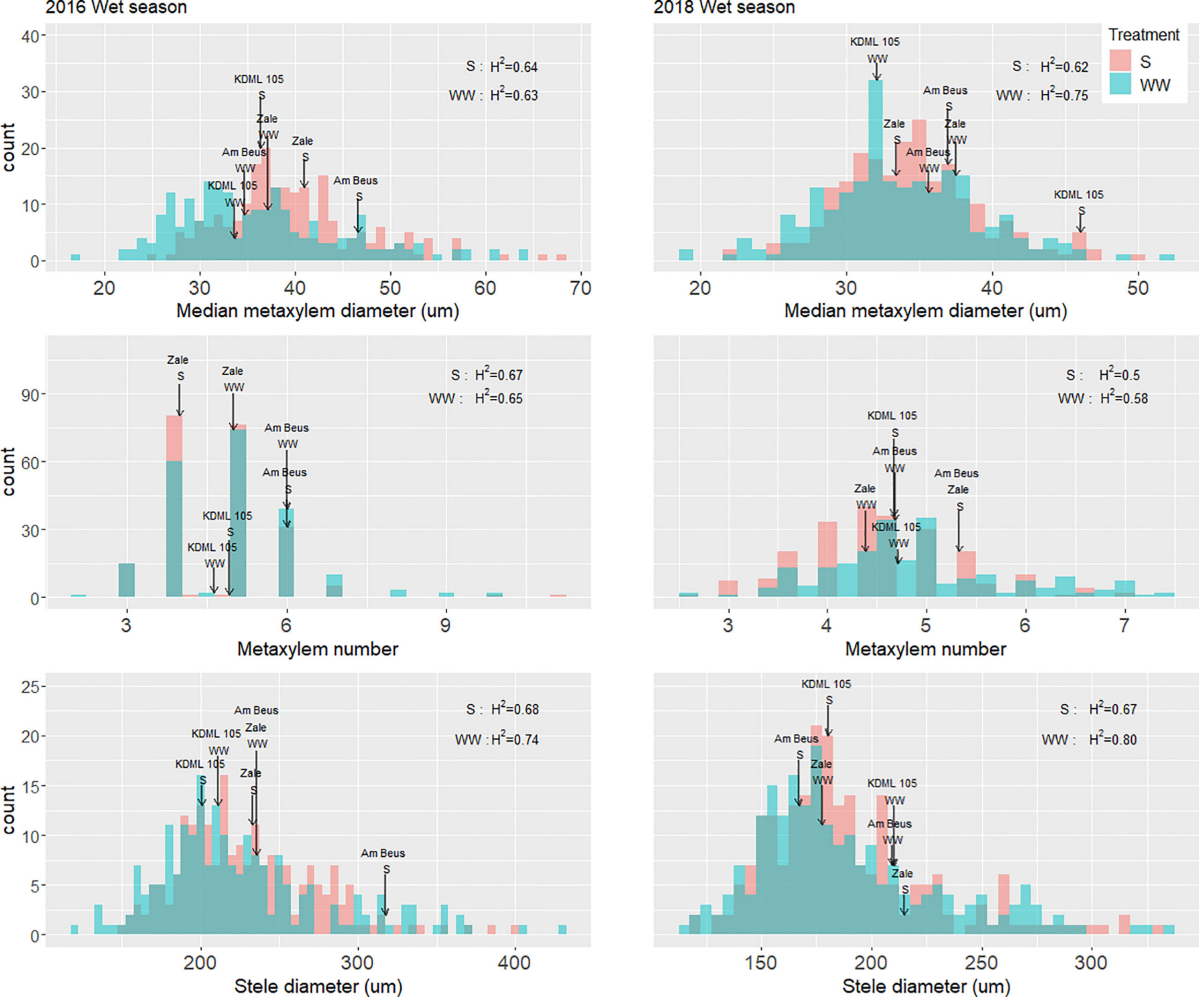
Distributions of root anatomical phenotypes in puddled, transplanted (2016WS, left) and direct seeded (2018WS; right) trials on the Thai/SE Asia panel characterized in this study. Arrows indicate the values for the check variety KDML 105 in each treatment, as well as for Am Beus and Zale which stood out in the accession rankings and AMMI analysis for grain yield (**Figure S5**). WS, wet season; S, drought stress treatment; WW, well-watered treatment; H^2^, broad-sense heritability.

A large degree of variation in shallow-root phenotypes was observed (**Figure 1**). In the experiments on the full set of accessions, total crown root number and crown root number per tiller were reduced in 2016 by 21 and 33%, respectively. A greater reduction in total crown root number was found in 2018 although there were some rice accessions with higher crown root number per tiller under drought stress than in well-watered conditions (**Figure S3**). There was a 17% increase in median metaxylem diameter in 2016 while a slight increase (3%) was observed in 2018 for the same phenotype. No difference in metaxylem number was observed in the two years. A slight increase (2%) in stele diameter was observed under drought in 2016 while no difference was found in 2018 (**Figure 2**). Among anatomical traits, H^2^ was highest for stele diameter, ranging from 0.67-0.8 (**Figure 2**). Between the shallow and the deep root architectural phenotypes, shallow nodal root, S and L-type lateral root lengths showed a more pronounced decrease than those of deep roots. Declines in shallow roots ranged from 61 to 73% while those in deep roots ranged from 11 to 25%, with S-type lateral roots more affected than the other types. L-type lateral roots was the least affected in both shallow and deep soil layers (**Figure S4**). In the experiments on the full sets as well as the smaller subsets, root phenotypes did not generally show a consistent response to drought as indicated by both positive and negative mean reduction values across experiments (**Table S2**), suggesting genetic variation in the response to drought among root phenotypes.

Based on the lsmeans of yield under drought on the full set of accessions in 2016 and 2018, we ranked each accession for yield under drought stress. In both years, one accession – Am Beus – ranked in the top 20 accessions for yield under drought. Of the root anatomy phenotypes, Am Beus stood out for high metaxylem number under drought in both seasons, and for large xylem diameter and stele diameter in 2016 (**Figure 2**). In an AMMI analysis on the 200 accessions across all four trials to identify the most stable and high yielding accessions across different conditions, the accession Zale (G205) stood out as being high and stable-yielding (**Figure S5**). Zale stood out for high metaxylem diameter in 2016 and high metaxylem number under drought in 2018 (**Figure 2**).

### 3.2 Phenotypic correlations

We aimed to identify root phenotypes that were most consistently related to grain yield and biomass under drought. In terms of direct correlations with all root phenotypes measured on the full set of accessions, only stele diameter reduction (the decrease in stele diameter under drought as compared to that in the well-watered treatment) was significantly negatively correlated with biomass in both seasons (**Figure S6**).

Across experiments with the smaller sets of accessions, we used PCA regression to identify root phenotypes that were most related to grain yield under drought stress. In multiple regression models following PCA across seasons using GY under drought stress as the independent variable, principal components beyond PC10 were significant; therefore we considered the phenotypes with the highest loading values in the first four PCs only. Eleven phenotypes showed the highest loading values in the first four PCs (**Table S3**). Based on the sign of the coefficient in the multiple regression model (**Table S4**) and the direction of the loading value within each PC, we estimated the relationship between these phenotypes and grain yield under drought stress to be negative for stele diameter, metaxylem number, average density, and shallow L-type lateral root length plasticity. We estimated the relationship with grain yield under drought stress to be positive for shallow L-type length, shallow nodal root length, shallow S-type lateral root length, deep S-type lateral root length, deep nodal root length, and percent deep root increase.

**Figure 3:**
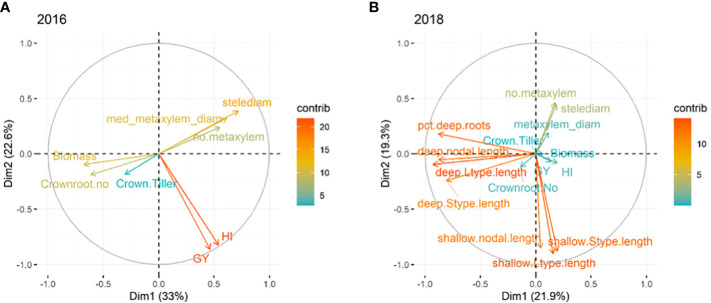
Principal components analysis of phenotypes in **(A)** agronomic, root anatomical and morphological phenotypes in puddled, transplanted (2016WS; left) and **(B)** direct seeded (2018WS; right) trials on the Thai/SE Asia panel. WS, wet season.

The experiments on the full set of accessions were subjected to principal component analysis. In 2016, the first three components in the PCA with Eigen value >1 contributed 72% of the total variation under drought stress. Five phenotypes contributed in PC1 accounting for 33.4% of the variation and two phenotypes contributed in PC2 accounting for 22.3%. PC3 with just one phenotype contributed 16.2% of the variation. All root anatomical phenotypes contributed positively in PC1 while biomass (0.66) and total crown root number (0.62) contributed negatively in PC1. Both grain yield (0.86) under stress and harvest index (0.81) had negative contributions in PC2, while crown roots per tiller had a positive contribution in PC3 (0.69) (**Figure 3A**). For 2018, the first five components with Eigen value >1 were considered in the PCA and the total contribution to the variation was 78.2%. PC1 included deep L-type lateral (0.74), nodal root length (0.74), and percent total deep length (0.83) which accounted for 23.7% of the variation. PC2 which contributed 19.4% of the variation was comprised of all shallow root phenotypes and deep S-type lateral root length (0.64) while PC3 contributed 13.3% and was comprised of grain yield (0.78), biomass (0.53) and harvest index (0.77). While all first three PCs contributed negatively to the phenotypes, PC4, which was comprised of all root anatomical phenotypes, contributed 12.6% and PC5 with root morphological phenotypes (crown root phenotypes) contributed 9.1%, all in the positive direction (**Figure 3B**).

**Figure 4:**
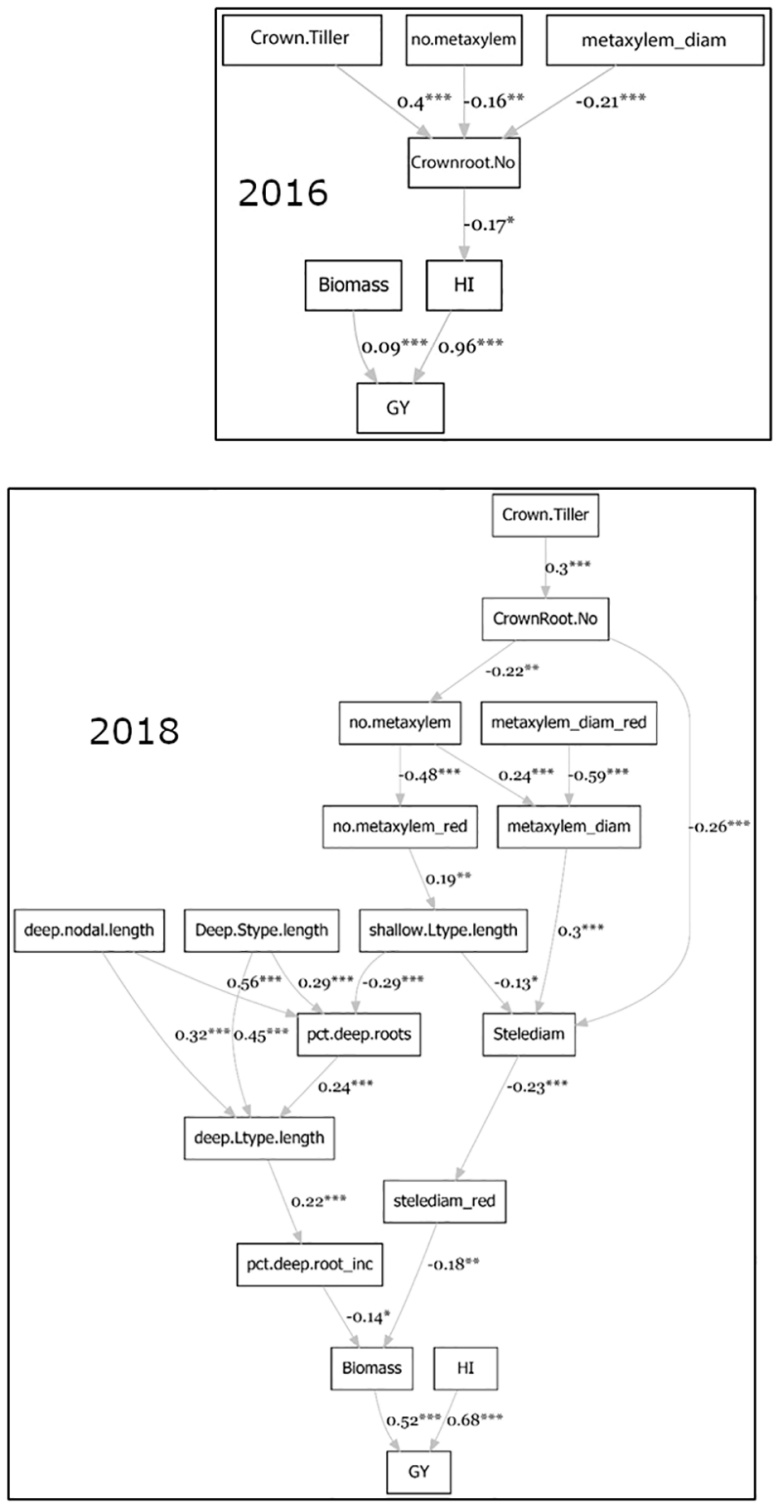
Path coefficient analysis on agronomic, root anatomical and morphological phenotypes in puddled, transplanted (2016WS; top) and direct seeded (2018WS; bottom) trials on the Thai/SE Asia panel. WS, wet season.

Path coefficient analysis (**Figure 4**) indicated that the phenotypes directly contributing to grain yield under stress were biomass and harvest index. The number of crown roots, metaxylem vessels, and the median metaxylem diameter indirectly contributed to grain yield under stress in both seasons. In 2018, the length of different root types (nodal, L-type lateral and S-type lateral) from the deep portion all contributed to grain yield through biomass. Stele diameter indirectly contributed (negatively) to grain yield through biomass in 2018.

### 3.3 Genetic analysis

#### 3.3.1 Linkage disequilibrium and population structure

In order to identify genetic regions related to the agronomic and root phenotypes measured by genome-wide association studies (GWAS), we first examined linkage disequilibrium and population structure on the full set of accessions grown in 2016 and 2018. Chromosome 6, followed by chromosomes 4 and 11, had the highest reduction in linkage disequilibrium (**Figure S7**). Taking the average across all chromosomes, LD r^2^ decayed to 0.1 at 360 kb and to r^2^ of 0.2 at 90 kb. We defined the LD distance specific for the analysis of this panel as the distance where LD r^2^ drops to 0.2 (i.e. 90kb). Matching the genotype and phenotype data resulted in a set of 209 varieties comprised of mainly two groups of sub-populations: indica and non-indica as indicated by a phylogenetic tree (**Figure S8**). The non-indica subpopulation was filtered out, resulting in a set of 164 accessions to be used for GWAS.

#### 3.3.2 GWAS analysis and identification of candidate genes

**Figure 5:**
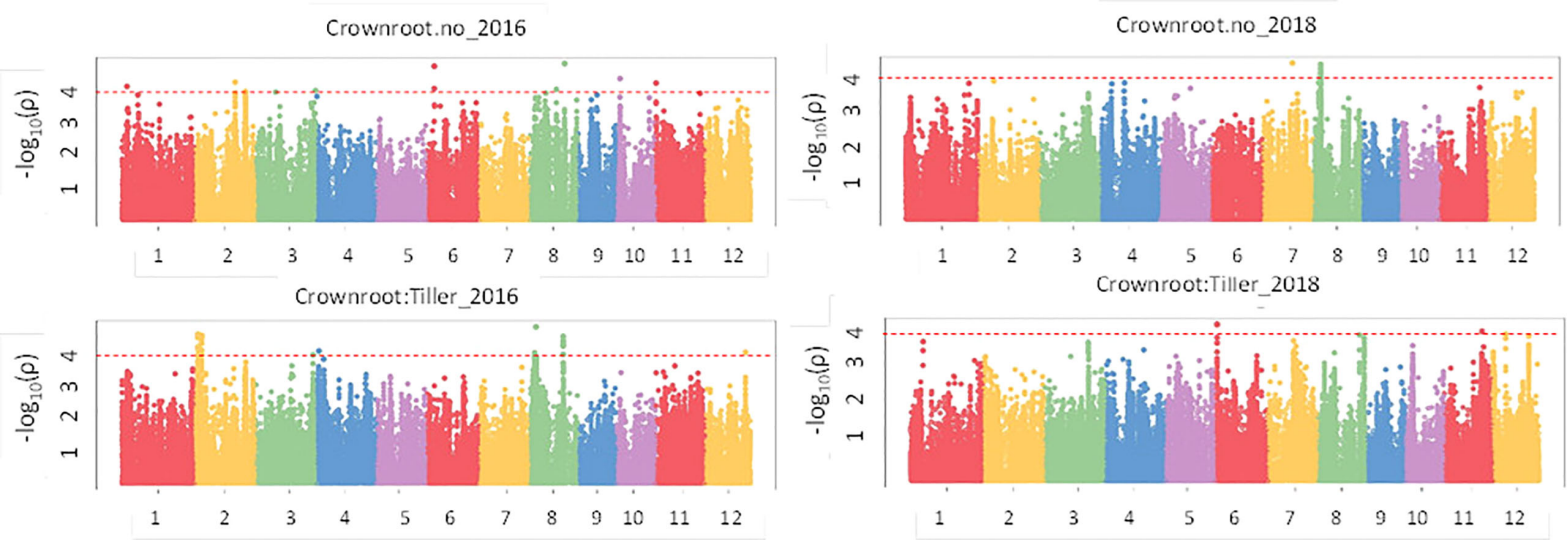
GWAS results for crown roots in puddled, transplanted (2016WS; left) and direct seeded (2018WS; right) trials on the Thai/SE Asia panel. WS, wet season.

**Figure 6:**
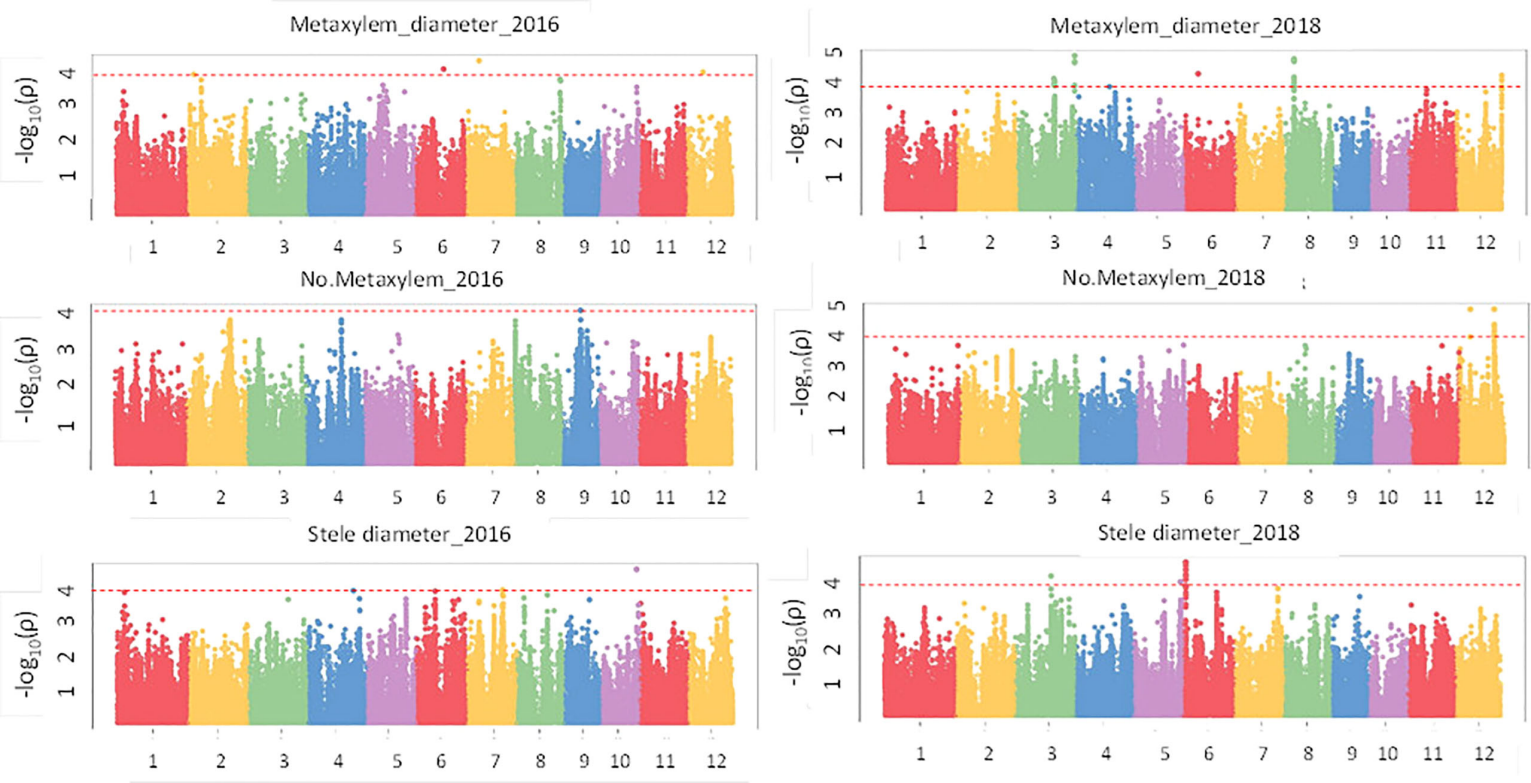
GWAS results for root anatomical phenotypes in puddled, transplanted (2016WS; left) and direct seeded (2018WS; right) trials on the Thai/SE Asia panel. WS, wet season.

Results of the association analysis for all phenotypes performed using the MLM method are presented in **Table S5**. There were 61 significant associations (p value< 1e-04) that were detected for crown root phenotypes (**Figure 5**) in 2016 and 2018 of which 50 had a p value< 1e-04 and 11 had a p value< 1e-05 and were distributed on all 12 chromosomes. The phenotypic variation explained (PVE) ranged from 8.5% to 27.1%. In the case of the root anatomical phenotypes (**Figure 6**) in 2016 and 2018, a total of 79 significant SNPs were associated with the phenotype. The PVE was between 10.4% to 19.8% where the maximum PVE was found in metaxylem number in 2018. Out of 79 SNPs associated, 60 were significant at p value< 1e-04 and 19 loci were significant at< 1e-05. There were 112 significant associations for root architectural phenotypes in 2018 (**Figure S9**). Out of 112 there was one locus with p value< 1e-06, 34 loci with p value< 1e-05 and 77 loci with p value< 1e-04. The deep S-type lateral root length had the highest number of significant loci (39). The PVE for root architectural phenotypes ranged from 10.7% to 24.4% with the maximum PVE found in deep L-type lateral root length.

Co-locations of GWAS QTL were found among phenotypes and between the two seasons for the same phenotype (**Table S6**). Phenotypes such as total crown root number, crown root number per tiller and stele diameter showed co-locations between experiments (2016WS and 2018WS) and were found on chromosomes 1, 3, 4, 6, 8, 11 and 12. Moreover, co-locations of QTL among root morphology, architecture and anatomy phenotypes were observed on all chromosomes except for chromosome 10 signifying no possible interactions among loci for root phenotypes in this region of the genome. Regions within 23.8 Mbp on chromosome 2, 2.4 and 14.6 Mbp on chromosome 5, 22 and 23.1 Mbp on chromosome 6 and 19.9 and 24.3 Mbp on chromosome 12 showed co-locations for crown root phenotypes, metaxylem phenotypes, stele diameter and length of lateral and nodal roots in the deeper soil portion. These regions of the genome are probably involved in root function under drought stress.

Significant GO terms for most phenotypes were generally listed as gene sets for biological processes, but those for stele diameter showed more diverse descriptions including cellular and metabolic processes and those related to nitrogen and phosphorus metabolism (**Table S7**). Gene Ontology (GO) enrichment analysis is a process for comparison of the curated annotations of particular subsets of gene models (co-located, expressed, in common to a study, etc.) to identify those that share ontology terms in common at a higher frequency than expected as compared to the whole genome. In this study, GO enrichment analysis indicated that the root phenotypes in this study are in general controlled by genes involved in cellular and metabolic processes, biological regulation and response to stimulus. A basic helix-loop-helix transcription factor classified under biological regulation and a non-specific phospholipase C3 were controlling crown root number. A gene related to median metaxylem diameter was found: the basic helix-loop-helix transcription factor controlling biological regulation. A metabolic process gene encoding carotenoid cleavage dioxygenase and gibberellin 2-beta-dioxygenase 8 was found for stele diameter. Moreover, a brassinosteroid-signaling kinase as response to stimulus was also found for stele diameter. Genes for metabolic processes were found controlling root length at shallow soil layers which include the cell wall associated receptor kinase 112 and the glycerol-3-phosphate acyltransferase. Likewise, the length of the roots at shallow soil layers was also controlled by NITRILASE1 as a stimulus affecting root length. On the other hand, genes controlling metabolic and cellular processes were found to control root length at deeper soil layers (**Table S8**).

Candidate genes were identified using Rice Annotation Project Database (RAP-DB) (Sakai et al., 2013) and significant GWAS SNPs were annotated by adding 100 kb upstream and downstream of the significant SNP. Functions of the candidate genes include transcription factors, transporters, receptors and catalase. The number of annotated genes for each QTL ranged from 13 (metaxylem number 2016) to 48 (deep S-type lateral root length) (**Table S9**). Genes containing functional SNPs that cause amino acid changes are listed in **Table 2**. Annotated genes having functions as transcription factors include Os06g0184000, Os06g0496400, Os05g0566800, Os06g0127100, Os03g0181600 while transporter genes include Os07g0232900, Os06g0128300. Other genes containing functional SNPs included catalase genes such as Os11g0593000, Os04g0550600, Os05g0560900, Os10g0180800 and receptor gene Os02g0640500.

**Table 2:** Candidate genes containing functional SNPs from the GWAS analysis on root phenotypes of the Thai/Southeast Asian panel.

Trait	Chrom	*Gene name*	*Gene Symbol*	Gene position	RAP ID
Crownroot.no_2016	6	*ROOTHAIRLESS1*	*OsbHLH115, OsRHL1*	4192207 - 4194418	Os06g0184000
Crownroot.no_2018	no candidate gene				
Crown.Tiller_2016	2	*Histone acetyltransferase HAC703*	*OsHAC703, OsKIX_2, OsHAC1*	1991843 - 1997302	Os02g0137500
	2	*Vacuolar cation/proton exchanger 2*	*OsCAX2, OsCAX4, OsSTA47, OsNCX3*	2073173 - 2075673	Os02g0138900
	2	*Phosphate Starvation Response3*	*OsPHR3, Os-PHR3, OsPHL1, PHL1*	2075629 - 2079136	Os02g0139000
	2	*Squamosa promoter-binding-like protein 3*	*OsSPL3*	2105726 - 2110544	Os02g0139400
	8	*plant U-box-containing protein 5*	*OsPUB5*	19875348 - 19881382	Os08g0415600
Crown.Tiller_2018	11	*Non-specific phospholipase C3*	*OsNPC4, OsNPC3*	22569981 - 22576763	Os11g0593000
Metaxylem_Diamter_2016	6	*ROOT HAIR DEFECTIVE-SIX LIKE 3*	*OsbHLH127, OsRSL3*	17348908 - 17350328	Os06g0496400
	7	*heavy metal ATPase 3*	*OsHMA3*	7405745 - 7409553	Os07g0232900
Metxylem_Diameter_2018	3	*Serine/threonine protein kinase*	*OsSAPK10, OsSnRK2.10*	23068746 - 23071156	Os03g0610900
	3	*Acyl-CoA-binding protein*	*OsACBP6*	35105148 - 35112495	Os03g0835600
	12	*Similar to Na+/H+ antiporter.*	*OsSOS1, OsNHA1*	27495775 - 27508468	Os12g0641100
No.Metaxylem_2016	no candidate gene				
No.Metaxylem_2018	12	*Diacylglycerol kinase 8*	*OsDGK8*	6729703 - 6731831	Os12g0224000
	12	*Respiratory Burst Oxidase Homolog H*	*OsrbohH*	21648123 - 21653555	Os12g0541300
Stele_Diameter_2016	4	*GATA transcription factor 21*	*OsGATA21*	27260363 - 27264477	Os04g0544500
	4	*Carotenoid cleavage dioxygenase*	*OsCCD7*	27567824 - 27570449	Os04g0550600
	10	*Brassinosteroid-signaling kinase*	*OsRLCK304, OsBSK1-2*	21199763 - 21204782	Os10g0542800
Stele_Diameter_2018	5	*gibberellin 2-beta-dioxygenase 8*	*OsGA2ox8*	27910754 - 27912924	Os05g0560900
	5	*Cold acclimation protein 413-TM1*	*Oscor413-tm1*	28213968 - 28216045	Os05g0566800
	6	*Dehydration-responsive element-binding protein 1C*	*OsDREB1C, OsERF026*	1434770 - 1435533	Os06g0127100
	6	*ABC transporter superfamily ABCB subgroup member 23*	*OsABCB23, OsISC32, OsATM3*	1492909 - 1502583	Os06g0128300
Shallow.Stype.Length_2018	1	*WALL-ASSOCIATED KINASE GENE 5*	*OsWAK5*	14809676 - 14821941	Os01g0363900
	2	*Salt Intolerance 1*	*OsSIT1*	25723956 - 25726080	Os02g0640500
	10	*cell wall associated receptor kinase 112*	*OsWAK112*	5539743 - 5544578	Os10g0180800
Shallow.Ltype.Length_2018	2	*phytosulfokine receptor 1*	*OsPSKR1, OsPSKR10*	25176174 - 25179701	Os02g0629400
	2	*GA 2-oxidase 9*	*OsGA2ox9*	25199505 - 25203742	Os02g0630300
	2	*NITRILASE1*	*OsNIT1*	25459520 - 25462586	Os02g0635000
	2	*Nitrilase 2*	*OsNIT2*	25465734 - 25469526	Os02g0635200
	2	*Salt Intolerance 1*	*OsSIT1*	25723956 - 25726080	Os02g0640500
Shallow.Nodal.Length_2018	1	*quiescent-center-specific-homeobox (QHB) gene*	*OsWOX9*	36813736 - 36815023	Os01g0854500
	1	*glycerol-3-phosphate acyltransferase*	*GPAT*	36863177 - 36871094	Os01g0855000
	1	*auxin transporter 1*	*OsLAX1, OsRAU1, OsAUX1*	36998338 - 37004643	Os01g0856500
	2	*Salt Intolerance 1*	*OsSIT1*	25723956 - 25726080	Os02g0640500
	11	*Rice WRKY gene61*	*OsWRKY61, OsWRKY103*	27740507 - 27741184	Os11g0685700
	11	*METALLOTHIONEIN I-1A*	*OsMT1a*	28827746 - 28828439	Os11g0704500
Deep.Stype.Length_2018	6	*CBL-interacting protein kinase 25*	*OsCIPK25*	20490388 - 20492271	Os06g0543400
	6	*Glutamate receptor homolog 3.5*	*OsGLR3.5*	28345366 - 28350758	Os06g0680500
	9	*Rice WRKY gene62*	*OsWRKY62*	14992918 - 14994888	Os09g0417800
	9	*CBL-interacting protein kinase 16*	*OsCIPK16*	15009182 - 15010837	Os09g0418000
	11	*With No Lysine kinase 6*	*OsWNK6*	2925715 - 2929173	Os11g0160300
	11	*b-ZIP transcription factor 81*	*OsbZIP81*	2939260 - 2942822	Os11g0160500
Deep.Ltype.Length_2018	3	*tubulin tyrosine ligase-like 12 protein*	*OsTTLL12*	4150750 - 4157419	Os03g0179000
	3	*GATA transcription factor 22*	*OsGATA22*	4272872 - 4277466	Os03g0181600
	3	*ABC transporter superfamily ABCB subgroup member 12*	*OsABCB12*	4279300 - 4281410	Os03g0181675
	3	*ABC transporter superfamily ABCB subgroup member 12*	*OsABCB12*	4281418 - 4284885	Os03g0181750
	3	*APETALA2/ethylene-responsive element binding protein 125*	*OsAP2-125*	4348736 - 4350531	Os03g0183000
	6	*Dehydration-responsive element-binding protein 1C*	*OsDREB1C*	1434770 - 1435533	Os06g0127100
	6	*GRAS protein 32, SMALL ORGAN SIZE 2*	*OsGRAS32*	1465500 - 1468583	Os06g0127800
	6	*ABC transporter superfamily ABCB subgroup member 23*	*OsABCB23*	1492909 - 1502583	Os06g0128300
	6	*ACC SYNTHASE 6*	*OsACS6*	1629778 - 1633507	Os06g0130400
Deep.Nodal.Length_2018	6	*class-1 type histone deacetylase 1*	*OsHDAC1*	22794359 - 22800197	Os06g0583400
Pct.Deep.roots_2018	7	*equilibrative nucleoside transporter*	*OsENT2*	22231027 - 22234551	Os07g0557100
	7	*equilibrative nucleoside transporter 3*	*OsENT3*	22236821 - 22240319	Os07g0557200
	7	*equilibrative nucleoside transporter 4*	*OsENT4*	22243925 - 22246490	Os07g0557400
	7	*F-box protein 396*	*OsFbox396*	22411227 - 22413069	Os07g0561300
	8	*Protein kinase (Leucine-rich repeat receptor like kinase)*	*SHR5*	6015745 - 6023225	Os08g0203400
	8	*Antioxidant Protein1, Heavy metal-associated protein 37*	*OsATX1, OsaATX1, OsHMP37*	6159123 - 6161383	Os08g0205400
	8	*Nuclear factor Y C5 subunit*	*OsHAP5F*	6212073 - 6217326	Os08g0206500
	8	*zinc transporter 4*	*OsZIP4*	6267823 - 6270904	Os08g0207500

GWAS was conducted on indica accessions grown in the experiments with the full set of accessions (2016WS and 2018WS).

### 3.3.2 SNP effects

**Figure 7:**
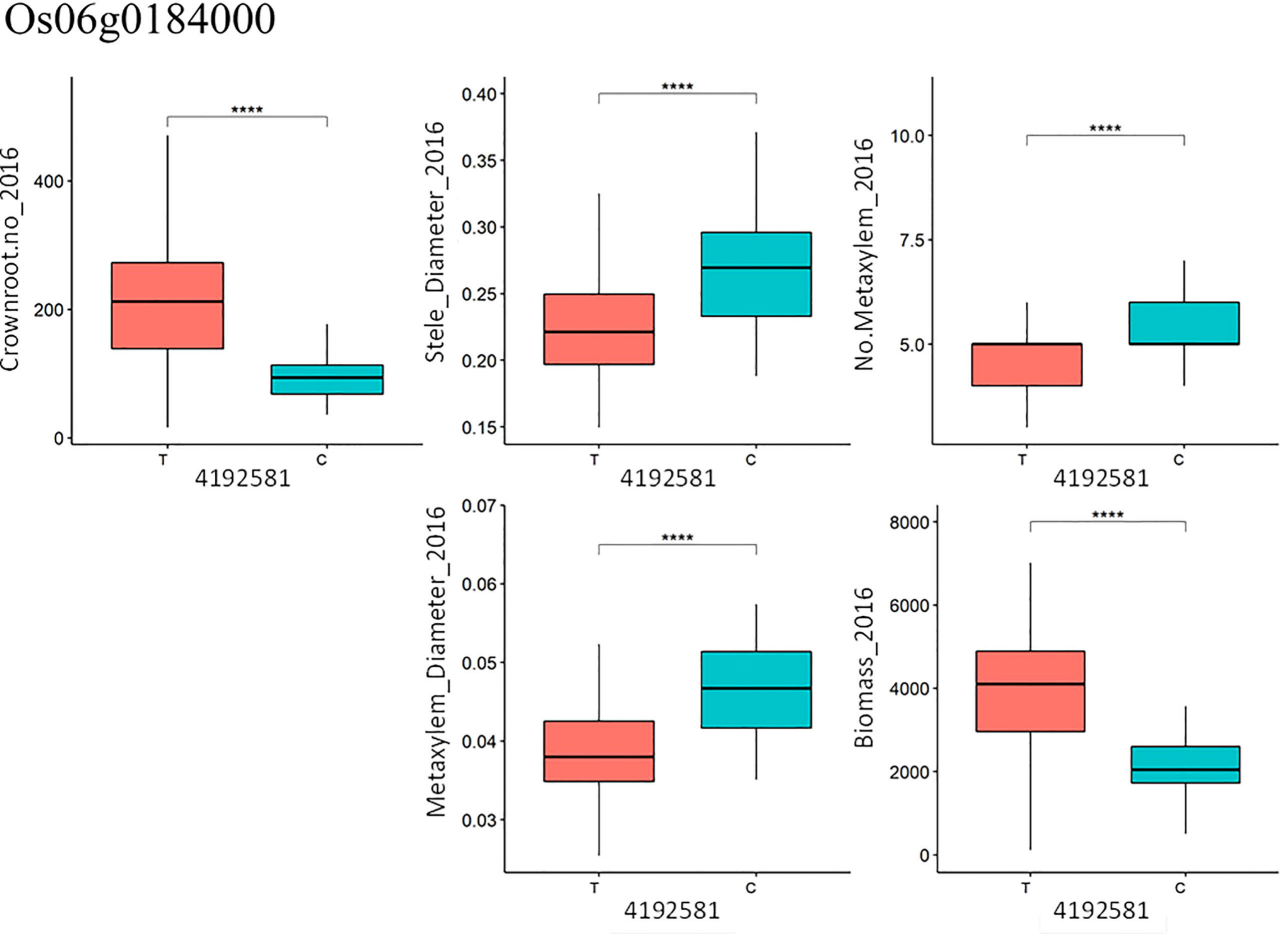
Functional SNPs at position 4192581 with T/C allele in gene Os06g0184000 (*OsRHL1*) for total crown root number (Crownroot.no_2016) and association with other phenotypes. **** denotes P<0.0001 based on t-test with Bonferroni correction.

From the candidate genes identified, functional SNPs causing amino acid changes were identified and the alleles were compared with the phenotype data. In the case of gene Os06g0184000 (*OsRHL1*), one SNP (T/C) at position 4192581 on chromosome 6, explaining 11.4% of the variation in total crown root number in 2016, was found to be significant and highly associated with the phenotype (**Figure 7**): accessions carrying allele T in gene Os06g0184000 had significantly higher total crown root number than those carrying allele C (**Figure 7**). In the case of median metaxylem diameter, there were four SNPs in gene Os06g0496400 (*OsRSL3*) that are significantly associated with the phenotype. SNP positions 17349241, 17349289, 17349299 and 17349635 contributed 28.4%, 2.9%, 8.4% and 30.3%, respectively to phenotypic variation in median metaxylem diameter in this panel (**Table S9**, **Table S10**, **Figure S10**). Another gene, Os07g0232900, also for median metaxylem diameter has one functional SNP at position 7407112 and contributed 26.7% to the variation found for the phenotype (**Table S9, Table S10, Figure S10**). Multiple regression was conducted using the SNPs for crown root number from all lines as the x value and the phenotypic value (for example, biomass) as the y value (**Table S11**). This multiple regression determined the significance of the relationships of total crown root number with stele diameter, metaxylem number, median metaxylem diameter and biomass. An increase in crown root number was correlated with the increase in biomass of the accessions tested (**Figure 7**). In contrast, metaxylem number, the median metaxylem diameter and stele diameter showed a negative relationship with biomass (**Figures 7**). Increased median metaxylem diameter resulted in decreased biomass and crown root number, and an increase in stele diameter and metaxylem number also resulted in decreased biomass (**Figure S10, Table S11**). Therefore, in the Thai/Southeast Asian rice panel used in this study, greater numbers of crown roots with small stele and xylem phenotypes were more favorable under drought stress.

## 4 Discussion

Among crop species, a range of root phenotypes have been reported as beneficial to productivity under drought stress, with deep root growth being the most consistently beneficial phenotype. The role of shallow root phenotypes such as crown root growth and root anatomy have been less frequently related to performance under drought. In this study, we characterized the shallow root growth of a panel of Thai/Southeast Asian rice accessions, in addition to characterizing root architecture across soil depths, in order to examine root phenotypes that might be affecting productivity under drought. Our results suggest that there are important components of both deep and shallow root growth behind drought response, as indicated in the PCA regression (**Tables S3 and S4**) as well as the PCA (**Figure 2**).

The deep root growth response was consistently observed under drought stress in this study, as evidenced by the shift in distributions under drought in the full set of accessions (**Figure S4**) as well as the increase in percent deep roots in experiments with the smaller subsets (**Table S2**). Few direct correlations between deep root growth and productivity were observed in this study, but this may be due to the very small root length values from depths of 30-60 cm in the soil core measurements conducted. In comparison, some of the shallow root phenotypes showed more frequent relationships with productivity under drought – especially in terms of biomass. Specifically, the stele diameter stood out across approaches. Indications of a large stele diameter as beneficial under drought include a) the large stele diameter of accession Am Beus (**Figure 2**), which ranked in the top 20 for grain yield in all four of the trials on the full set of accessions, and b) the direct negative correlation between biomass under drought and stele diameter reduction (**Figure S6**). Indications of a smaller stele diameter as beneficial under drought include a) the negative relationship of grain yield and stele diameter in the PCA regression (**Tables S3 and S4**), b) the negative relationship between stele diameter and biomass (thus indirectly affecting grain yield) in the 2018 path coefficient analysis (**Figure 4**), c) the opposite trend of stele diameter and grain yield under drought in the 2018 PCA (**Figure 3**), and d) the opposite trends of stele diameter and biomass under drought based on SNP effect (**Figure 7** and **S10**). Given the inconsistent relationships between stele diameter and productivity under drought in this study, future studies across more environments might help dissect the specific environmental conditions or other root phenotypes that are affecting these relationships.

Stele size is mainly a function of the number and size of the vascular elements. In this study, stele diameter was consistently positively correlated with both metaxylem number and diameter (**Figure S6**). Stele diameter has also been reported to closely reflect the whole-root diameter (Uga et al., 2008, Jeong et al., 2013). However, drought stress may affect these relationships: Hazman and Brown (2018) observed rice stele diameter to be largely maintained under drought, at the expense of the size of the cortex. Overexpressing *OsNAC10* in the roots was reported to increase yield under drought and to be related to both whole-root and stele cross sectional size (Jeong et al., 2010). In a study conducted by Manikanta et al. (2022), drought tolerant lines N22, Karuthamodan and Chuvannamodan had increase in stele diameter, late metaxylem diameter and late metaxylem number whereas the susceptible lines Annapoorna, Jyothi and Swetha showed decreases in those root anatomical phenotypes. Kadam et al. (2015) concluded that the lack of variation in rice root anatomical phenotypes such as stele diameter may explain the poor productivity of rice under drought as compared to wheat, and they did not find differences in root, stele, or xylem diameter in rice response to drought stress, although the drought-tolerant variety N22 showed increased stele diameter near the root apex. Fonta et al. (2022b) reported temporal, water treatment, varietal, and depth of sampling effects on rice nodal root anatomy, and although drought-tolerant variety Azucena showed a larger stele area than drought-susceptible variety IR64, the deep (apical) stele area was more reduced by drought stress. In summary, these previous studies have generally concluded that a larger stele diameter was beneficial under drought stress. This is in contrast to the four examples (listed above) of a smaller stele diameter being beneficial under drought in the present study.

Other shallow root phenotypes in our study – crown root number and crown roots per tiller – were not consistently related to productivity under drought in this study. Moyers et al. (2022) reported crown root number to be correlated with growth and productivity under vegetative stage drought stress in the rice Global MAGIC population under dry direct-seeded conditions. Ruangsiri et al. (2021) found that crown root number was reduced in KDML 105 chromosome segment substitution lines when planted in clay soil, but an increase in crown root number was found in the same population when planted in sandy soil condition. Indeed, the environmental conditions including soil type and drought severity likely affect the relationship between many root phenotypes and productivity under drought.

At the phenotypic level, statistical analysis employing advanced clustering (e.g. Klein et al., 2020) and simulation modeling (e.g. Ajmera et al., 2022) can help improve our understanding of the interactions among root phenotypes and their combined effect on productivity under drought. Genetic correlations and candidate gene function may also help characterize correlations among phenotypes. In our study, since the detailed SNP data was available for all of the accessions on which root phenotypes were measured, we took the approach of identifying QTLs (GWAS peaks), underlying candidate genes, and SNP effects, in order to better understand the relationships among candidate genes involved in the phenotypes and their response to drought. Several markers were significantly associated with root phenotypes in the Southeast Asian rice accessions studied here. The differences in GWAS peaks and candidate genes that were identified for root traits in the 2016 and 2018 experiments despite similar soil moisture levels at the respective sampling dates reflect cumulative differences in environmental conditions across the two growing seasons. Co-locations of phenotypes that were identified in 2016 and 2018 were found for crown root number, crown root number per tiller and stele diameter which confirms the genetic control of those genomic segments in multiple environments. Moreover, co-location of QTL for different root phenotypes (morphology, anatomy, architecture) suggest pleiotropic effects, however, more analysis is needed to confirm it.

Similar candidate genes, namely *OsDREB1C* and *OsABCB23* were identified for stele diameter in 2018 and for deep L-type lateral root length on chromosome 6. *OsABCB23*, also known as *OsATM3*, has been reported to have an important role in the defense to oxidative stress and response to the stress in both leaves and roots (Liang et al., 2014). Oxidative stress severely inhibits root growth, and the presence of *OsATM3* has been reported as important in lateral root development and root tip growth and oxidative stress (Zou et al., 2017). The gene Os06g0127100, which is dehydration-responsive element-binding protein 1C (*OsDREB1C*), with a functional SNP at position 1,434,806 for stele diameter in 2018 explaining 16.7% of the phenotypic variation was significantly associated with metaxylem diameter, number of metxylem, biomass, and crown root number in 2018. This is an example of a QTL identified by GWAS and identified as a functional SNP. The expression of *OsDREB1C* is induced by light and low-nitrogen levels. Overexpression lines of *OsDREB1C (OsDREB1C-OE)* exhibited higher grain yield by increasing the grain number per panicle (Wei et al., 2022). Moreover, the increase in yield was due to improved nitrogen-use efficiency conferred by *OsDREB1C-OE* by stimulating nitrogen uptake of roots. *OsDREB1C-OE* lines were found to have longer roots (Wei et al., 2022). In a study conducted by Wang et al. (2022), *OsDREB1C* was found to control basal chilling tolerance in rice. In addition, *OsDREB1C* was found to positively control salt tolerance in rice (Wang et al., 2022). These genes as well as *OsRHL1* and *OsRSL3*, which have been reported to be involved in root hair development and whose allelic variation was correlated with performance under drought in this study, may represent the importance of overall root function under stress as well as the coinciding processes of water and nutrient uptake, root hair growth, and increased stele permeability in the most basal developmental zone of the root (Jones et al., 2009).

We detected several markers associated with root phenotypes, and dissected those phenotypes in indica accessions using GWAS analysis. The indica subset of our Thai/Southeast Asian panel had an average LD decay distance of 111kb which agrees with previous calculations (Mather et al., 2007; Lu et al., 2015). QTL for crown root number in 2018 located on chromosome 1 at position 35086871 and crown root number per tiller on chromosome 7 at position 15037756 were found to be co-located with crown root number phenotypes identified by Phung et al. (2016). Several root thickness QTL on chromosome 7 at position 15924916 and on chromosome 8 at position 4610655 identified by Phung et al. (2016) co-located with stele diameter QTL in the 2016 experiment from this study. Moreover, Phung et al. (2016) identified QTL positions on chromosome 4 at position 12548297 and on chromosome 6 at position 22370305 that co-located with shallow S-type lateral root and deep nodal root length, respectively. Additional QTL co-located with deep S-type lateral root length on chromosomes 1 and 4, located at 29660200 and 21337715 positions, respectively, that pertain to root dry mass below 30 cm soil depth and deep root biomass (Courtois et al., 2013).

Based on the gene ontology enrichment analysis, large numbers of genes for all root phenotypes were mainly associated with cellular and metabolic processes. In addition to the cellular and metabolic processes, biological regulation and response to stimulus were common biological processes specific to the crown root phenotypes. Garg et al. (2020) identified GO terms that are related with metabolic processes involved with genes that are induced during crown root primordia (CRP) growth while some genes involved in biological regulation and signaling were highly expressed in the post-embryonic root organogenesis since they were identified in the initiating CRP and their expression is reduced as the CRP grows. Gene enrichment analysis for candidate genes for root anatomical phenotypes such as stele diameter and median metaxylem diameter also identified genes involved in signaling, while the number of metaxylem involved only metabolic and cellular process and localization (as identified in the 2018 experiment). This suggests that there are fewer types of genes controlling the metaxylem number. Nodal root length and lateral root length from the shallow soil layer involved genes of different categories including responses to stimulus, biological regulation, signaling, developmental processes and multicellular organismal processes, while the same phenotypes under deep soil layers had the same gene control except for multicellular organismal processes and developmental processes. In summary, the favorable haplotypes identified for biomass in our SNP effect analysis suggested that, at least in our panel of Thai/Southeast Asian rice accessions, a smaller basal stele diameter may be beneficial under drought stress.

## 5 Conclusion

The relationship between stele diameter size and rice response to drought has been inconsistent in previous reports, as well as in the current study depending on the approach considered. The set of accessions studied, environment in which they were grown, and the location of root anatomy observations (basal vs apical) all likely affect this relationship. In this study focused on basal root anatomy of a Thai/Southeast Asian rice panel, the most consistent result across phenotypic and genetic approaches was a negative relationship between stele diameter and biomass and yield under drought. The current study emphasized the strength of a genetic analysis to provide insight on phenotypic relationships through SNP effects on various phenotypes. In addition to these implications for the phenotypic correlations, the favorable haplotypes identified may be further investigated for use in breeding. Our next step will be to characterize the frequency of these haplotypes in an elite pool of drought breeding lines, to assess if it would be beneficial to introgress them into an elite background for further testing and validation.

## Data availability statement

The datasets presented in this study can be found online on the IRRI Dataverse page: https://dataverse.harvard.edu/dataverse/RiceResearch.

## Author contributions

JS and BT, formal analysis, visualization, writing – original draft preparation. MN, data curation, investigation, visualization. MQ, data curation, formal analysis, visualization. DC, methodology, writing – review & editing. KM, resources, writing – review & editing. JL, funding acquisition, investigation, project administration, writing – review & editing. KB, investigation, writing – review & editing. AH, conceptualization, funding acquisition, project administration, writing – original draft preparation. All authors contributed to the article and approved the submitted version.

## Funding

This work was funded by the BBSRC Newton Fund Grant BB/N013697/1, as well as the Bill and Melinda Gates Foundation project OPP1088843.

## Acknowledgments

We thank Michael Williams, Robert Snyder, and Hannah Schneider for assistance with the laser ablation and anatomical analysis at PSU. The University of the Philippines Los Baños kindly allowed use of their critical point dryer for this study. We thank Rolando Torres, Leo Holongbayan, Carlo Cabral, Eleanor Mico, Lesly Navarro, Ariston Reyes, and Philip Zambrano for support in conducting the field trials and analyzing the root samples at IRRI, as well as Rose Imee Zhella Morantte and Mathurada Ruangsiri for conducting statistical analysis and Marjorie De Ocampo for support of the GWAS analysis.

## Conflict of interest

The authors declare that the research was conducted in the absence of any commercial or financial relationships that could be construed as a potential conflict of interest.

## Publisher’s note

All claims expressed in this article are solely those of the authors and do not necessarily represent those of their affiliated organizations, or those of the publisher, the editors and the reviewers. Any product that may be evaluated in this article, or claim that may be made by its manufacturer, is not guaranteed or endorsed by the publisher.
