# Supplementary material for: Response of Southeast Asian rice root architecture and anatomy phenotypes to drought stress
Source: Front Plant Sci. 2022 Oct 19;13:1008954. doi: 10.3389/fpls.2022.1008954 (PMC9629509; doi:10.3389/fpls.2022.1008954)
Supplement: Supplementary file 1 [file DataSheet_1.zip › Table S2.docx]

|  | **2017DS (early duration)** | | | | | | |  | **2017WS (medium duration)** | | | | | | | |  | **2018DS (late duration)** | | | | | |
| --- | --- | --- | --- | --- | --- | --- | --- | --- | --- | --- | --- | --- | --- | --- | --- | --- | --- | --- | --- | --- | --- | --- | --- |
|  | **Median** | | | **Mean** | | **Min** | **Max** |  | **Median** | | **Mean** | | **Min** | | **Max** | |  | **Median** | **Mean** | **Min** | | **Max** | |
| **GY (g m^-2^)** | | 29.40 | 34.15 | | 0.00 | | 103.89 |  | 11.78 | 34.10 | | 0.00 | | 192.52 | |  | | 14.31 | 26.46 | 0.00 | 92.33 | |  |
| **GY_red** | | 0.89 | 0.87 | | 0.60 | | 1.00 |  | 0.95 | 0.87 | | 0.33 | | 1.00 | |  | | 0.96 | 0.90 | 0.54 | 1.00 | |  |
| **Biomass (g m^-2^)** | | 154.54 | 158.06 | | 83.15 | | 307.41 |  | 387.06 | 371.00 | | 0.00 | | 1119.76 | |  | | 318.27 | 331.40 | 251.07 | 471.68 | |  |
| **HI** | | 0.16 | 0.18 | | 0.00 | | 0.39 |  | 0.03 | 0.05 | | 0.00 | | 0.22 | |  | | 0.05 | 0.07 | 0.00 | 0.21 | |  |
| **Crownroot #** | | 137.00 | 170.35 | | 41.00 | | 673.00 |  | 519.50 | 566.46 | | 33.00 | | 1258.00 | |  | | 335.50 | 346.89 | 158.00 | 529.00 | |  |
| **Crownroots per tiller** | | 33.75 | 36.29 | | 14.80 | | 81.00 |  | 34.91 | 37.97 | | 16.50 | | 74.69 | |  | | 31.36 | 31.31 | 18.93 | 49.56 | |  |
| **# metaxylem** | | 5.00 | 5.39 | | 3.00 | | 10.00 |  | 4.00 | 4.63 | | 2.00 | | 9.00 | |  | | 4.00 | 4.52 | 3.00 | 6.00 | |  |
| **# metaxylem_plas** | | 0.17 | 0.11 | | -0.88 | | 0.48 |  | 0.06 | 0.03 | | -0.59 | | 0.50 | |  | | 0.00 | -0.06 | -0.43 | 0.20 | |  |
| **stelediam (um)** | | 219.95 | 223.15 | | 134.00 | | 352.46 |  | 155.79 | 163.41 | | 108.57 | | 318.22 | |  | | 176.79 | 175.46 | 106.48 | 228.51 | |  |
| **stelediam_plas** | | -0.19 | -0.18 | | -0.95 | | 0.33 |  | 0.76 | 0.77 | | 0.67 | | 0.86 | |  | | -0.52 | -0.48 | -0.99 | 0.26 | |  |
| **med_metaxylem_diam (um)** | | 41.63 | 40.78 | | 25.33 | | 57.85 |  | 32.08 | 32.60 | | 19.31 | | 55.71 | |  | | 37.84 | 36.13 | 24.37 | 46.56 | |  |
| **med_metaxylem_diam_plas** | | 0.02 | 0.01 | | -0.37 | | 0.35 |  | -0.06 | -0.07 | | -0.82 | | 0.38 | |  | | -0.40 | -0.35 | -0.83 | 0.25 | |  |
| **deep.L.type.length (cm)** | | 55.76 | 60.51 | | 7.73 | | 194.76 |  | 65.92 | 78.01 | | 5.21 | | 273.38 | |  | | 50.26 | 77.16 | 1.37 | 302.89 | |  |
| **deep.L.type.length_plas** | | 0.63 | 0.86 | | -0.82 | | 4.12 |  | 1.30 | 1.87 | | -0.82 | | 9.59 | |  | | 0.43 | 1.05 | -0.97 | 5.46 | |  |
| **deep.nodal.length (cm)** | | 23.58 | 24.06 | | 5.88 | | 52.44 |  | 12.69 | 14.63 | | 0.00 | | 37.06 | |  | | 35.88 | 47.33 | 7.69 | 148.00 | |  |
| **deep.S.type.length (cm)** | | 29.88 | 34.75 | | 3.32 | | 102.77 |  | 27.05 | 44.34 | | 0.00 | | 286.47 | |  | | -0.45 | -0.40 | -0.95 | 0.36 | |  |
| **shallow.L.type.length (cm)** | | 42.08 | 47.22 | | 14.23 | | 90.73 |  | 82.85 | 103.19 | | 24.95 | | 309.47 | |  | | 364.19 | 359.56 | 151.50 | 622.91 | |  |
| **shallow.L.type.length_plas** | | -0.59 | -0.54 | | -0.92 | | 0.12 |  | -0.24 | -0.12 | | -0.72 | | 1.60 | |  | | -0.53 | -0.55 | -0.81 | -0.23 | |  |
| **shallow.nodal.length (cm)** | | 38.69 | 41.25 | | 18.14 | | 60.87 |  | 64.70 | 79.03 | | 25.63 | | 203.03 | |  | | 166.89 | 172.18 | 83.78 | 316.08 | |  |
| **shallow.S.type.length (cm)** | | 58.62 | 67.84 | | 12.63 | | 141.93 |  | 168.27 | 218.58 | | 29.85 | | 749.47 | |  | | 35.95 | 34.37 | 0.00 | 45.98 | |  |
| **% deep roots** | | 65.60 | 60.76 | | 23.98 | | 88.80 |  | 44.25 | 44.45 | | 13.05 | | 66.47 | |  | | 8.28 | 12.90 | 0.72 | 39.39 | |  |
| **% deep roots_inc** | | 42.05 | 38.56 | | -4.68 | | 64.49 |  | 3.20 | 3.12 | | -25.02 | | 34.59 | |  | | 5.98 | 9.40 | -3.70 | 37.67 | |  |
| **ANG_TOP_DS** | | 45.74 | 42.37 | | 2.49 | | 67.23 |  | 45.36 | 43.72 | | 10.76 | | 77.12 | |  | | 43.28 | 43.56 | 24.46 | 58.05 | |  |
| **ANG_TOP_plas** | | 0.06 | 0.09 | | -0.61 | | 0.95 |  | 0.03 | -0.02 | | -1.75 | | 0.74 | |  | | 0.01 | 0.02 | -0.34 | 0.45 | |  |
| **AVG_DENSITY_DS** | | 2.46 | 3.16 | | 0.81 | | 11.54 |  | 1.60 | 1.97 | | 0.67 | | 6.04 | |  | | 5.95 | 6.78 | 1.91 | 25.09 | |  |

**Table S2.** Descriptive statistics of the three field trials grown using smaller subsets of genotypes chosen for their similar phenology and contrasting drought response. Values shown are from the drought stress treatments, and for grain yield reduction and root trait plasticity in comparison with the well-watered treatment. Root length values indicate cm of root within the 0-30 cm (shallow) and 30-60 cm (deep) segments of the 4-cm diameter soil core.
