# Supplementary material for: Response of Southeast Asian rice root architecture and anatomy phenotypes to drought stress
Source: Front Plant Sci. 2022 Oct 19;13:1008954. doi: 10.3389/fpls.2022.1008954 (PMC9629509; doi:10.3389/fpls.2022.1008954)
Supplement: Supplementary file 1 [file DataSheet_1.zip › Table S3.docx]

**Table S3.** PCA regression loading values across the three seasons of experiments on smaller subsets. The gray shading indicates traits with the highest loading values in each principal component. B) Summary of step-wise multiple regression analysis following PCA across the three seasons of experiments on smaller subsets.

|  | PC1 | PC2 | PC3 | PC4 |
| --- | --- | --- | --- | --- |
| GY_red | -0.0903 | 0.1866 | 0.2818 | -0.0091 |
| Crownroot | -0.2863 | 0.0599 | 0.2222 | -0.1039 |
| Crownroot.Tiller | -0.0513 | 0.0141 | 0.1592 | 0.082 |
| AVG_DENSITY | -0.1483 | -0.3100 | 0.0138 | 0.0534 |
| ANG_TOP | -0.2571 | -0.0348 | -0.1188 | -0.2165 |
| ANG_TOP_inc | 0.0206 | 0.0809 | 0.3779 | 0.1097 |
| stelediam | 0.3021 | -0.113 | 0.2426 | 0.2582 |
| # metaxylem | 0.2741 | -0.0816 | 0.113 | 0.3286 |
| med_metaxylem_diam | 0.2493 | -0.1289 | 0.2185 | 0.2824 |
| stelediam_red | -0.0702 | 0.2956 | -0.3442 | 0.1433 |
| # metaxylem_red | -0.0425 | 0.2278 | 0.0626 | -0.3335 |
| med_metaxylem_diam_red | 0.1653 | 0.2942 | -0.0457 | -0.2803 |
| shallow.S.type.length | -0.2064 | 0.3629 | -0.0464 | 0.2633 |
| deep.S.type.length | -0.0882 | 0.3896 | 0.1168 | 0.1684 |
| shallow.L.type.length | -0.3231 | -0.2493 | 0.1161 | 0.0465 |
| deep.L.type.length | -0.2324 | 0.223 | 0.2588 | 0.1042 |
| shallow.L.type.length_plas | -0.1056 | 0.1345 | -0.2179 | 0.5025 |
| deep.L.type.length_plas | -0.1836 | 0.2179 | 0.1635 | 0.0799 |
| shallow.nodal.length | -0.3626 | -0.1527 | 0.1319 | 0.1047 |
| deep.nodal.length | -0.2189 | -0.1289 | 0.3257 | -0.0399 |
| % deep roots | 0.2783 | 0.2977 | 0.1226 | -0.0347 |
| % deep roots_inc | 0.2134 | 0.079 | 0.3687 | -0.2695 |
