# Supplementary material for: Response of Southeast Asian rice root architecture and anatomy phenotypes to drought stress
Source: Front Plant Sci. 2022 Oct 19;13:1008954. doi: 10.3389/fpls.2022.1008954 (PMC9629509; doi:10.3389/fpls.2022.1008954)
Supplement: Supplementary file 1 [file DataSheet_1.zip › Table S4.docx]

**Table S4.** Summary of step-wise multiple regression analysis following PCA across the three seasons of experiments on smaller subsets.

| LINEAR REGRESSION ANALYSIS | | | | |  |  |  |
| --- | --- | --- | --- | --- | --- | --- | --- |
| Model Fitted: GYstress_means ~ PC1 + PC2 + PC3 + PC4 + PC5 + PC6 + PC7 + PC8 + PC9 + PC10 + PC11 + PC12 + PC13 + PC14 + PC15 + PC16 + PC17 + PC18 | | | | | | | |
|  |  |  |  |  |  |  |  |
| Analysis of Variance Table | | | |  |  |  |  |
| ------------------------------------------------------------------------------- | | | | | |  |  |
| Source | DF | Sum of Square | Mean Square | F Value | Pr(>F) |  |  |
| ------------------------------------------------------------------------------- | | | | | |  |  |
| Model | 18 | 39494.84 | 2194.158 | 5128.16 | 0 |  |  |
| Error | 22 | 9.413 | 0.4279 |  |  |  |  |
| Total | 40 | 39504.26 |  |  |  |  |  |
| ------------------------------------------------------------------------------- | | | | | |  |  |
|  |  |  |  |  |  |  |  |
| Model Summary: | | |  |  |  |  |  |
| ------------------------------------------------------------------------------------ | | | | | | |  |
| Root MSE GYstress_means Mean CV(%) R-Square Adj R-Sq | | | | | | | |
| ------------------------------------------------------------------------------------ | | | | | | |  |
| 0.6541 31.10 101.03 0.9998 0.9996 | | | | | |  |  |
| ------------------------------------------------------------------------------------ | | | | | | |  |
|  |  |  |  |  |  |  |  |
| Parameter Estimates: | | |  |  |  |  |  |
| -------------------------------------------------------- | | | | | |  |  |
| Variable | Estimate | Std. Error | t value | Pr(>\|t\|) |  |  |  |
| ----------- | ----------- | ------------- | ---------- | ----------- |  |  |  |
| Intercept | 31.1 | 0.1022 | 304.48 | 0 |  |  |  |
| PC1 | -2.8 | 0.043 | -65.98 | 0 |  |  |  |
| PC2 | 5.86 | 0.0515 | 113.91 | 0 |  |  |  |
| PC3 | 8.85 | 0.0544 | 162.67 | 0 |  |  |  |
| PC4 | -0.3 | 0.0813 | -3.51 | 0.002 |  |  |  |
| PC5 | -2.1 | 0.0907 | -23.24 | 0 |  |  |  |
| PC6 | -9.2 | 0.1011 | -91.21 | 0 |  |  |  |
| PC7 | 12.8 | 0.1051 | 121.33 | 0 |  |  |  |
| PC8 | -11 | 0.117 | -94.45 | 0 |  |  |  |
| PC9 | 9.98 | 0.1267 | 78.78 | 0 |  |  |  |
| PC10 | -8.3 | 0.1404 | -59.43 | 0 |  |  |  |
| PC11 | 6.39 | 0.1654 | 38.64 | 0 |  |  |  |
| PC12 | -4.8 | 0.1743 | -27.68 | 0 |  |  |  |
| PC13 | 1.15 | 0.2027 | 5.69 | 0 |  |  |  |
| PC14 | -13 | 0.23 | -56.31 | 0 |  |  |  |
| PC15 | 6.77 | 0.2648 | 25.56 | 0 |  |  |  |
| PC16 | -1.6 | 0.2917 | -5.32 | 0 |  |  |  |
| PC17 | 4.67 | 0.4439 | 10.51 | 0 |  |  |  |
| PC18 | -1.6 | 0.457 | -3.51 | 0.002 |  |  |  |
| ----------- | ----------- | ------------- | ---------- | ----------- |  |  |  |
