# Supplementary material for: Response of Southeast Asian rice root architecture and anatomy phenotypes to drought stress
Source: Front Plant Sci. 2022 Oct 19;13:1008954. doi: 10.3389/fpls.2022.1008954 (PMC9629509; doi:10.3389/fpls.2022.1008954)
Supplement: Supplementary file 1 [file DataSheet_1.zip › Supp Figs.docx]

***Supplemental Figures***


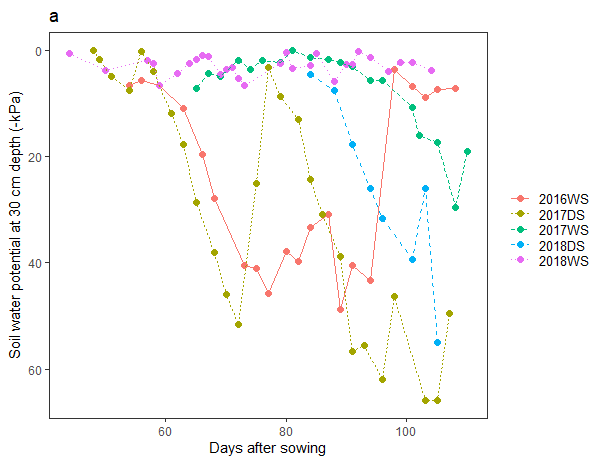


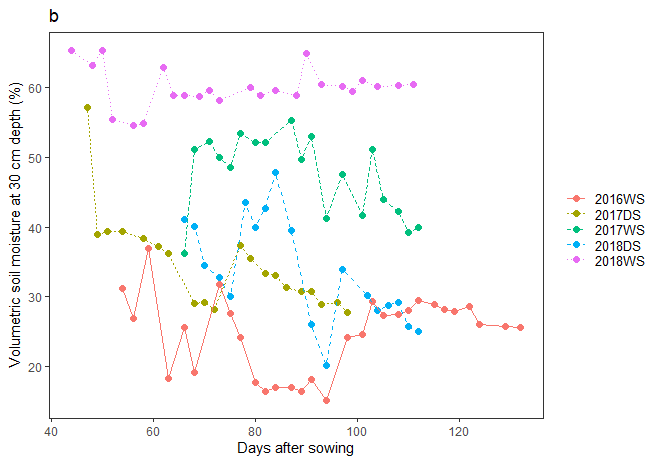


**Figure S1.** Soil moisture levels at a depth of 30 cm in the drought stress treatments of this study, as shown by a) soil water potential and b) volumetric soil moisture.


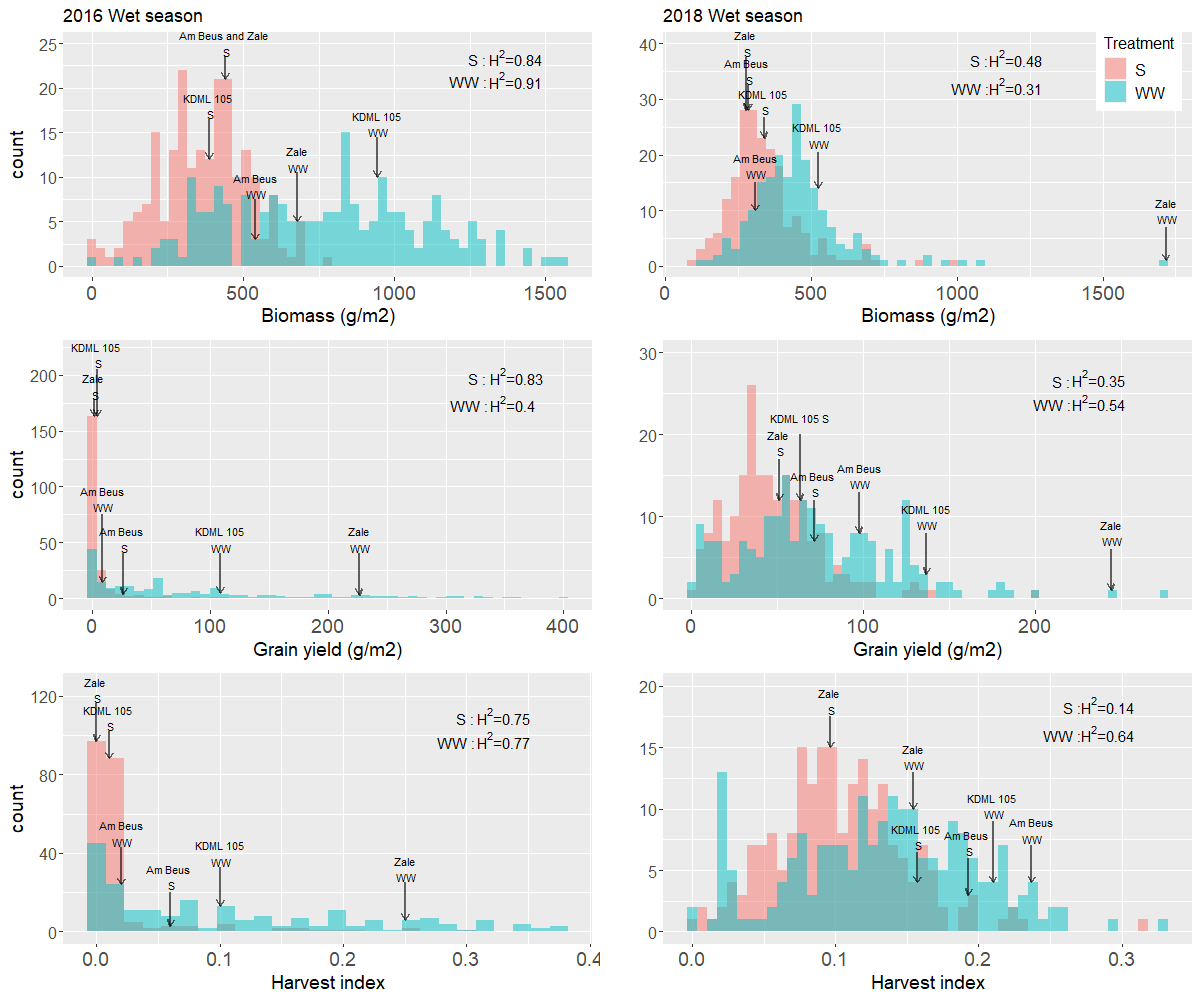


**Figure S2.** Distribution of agronomic traits in the puddled, transplanted (2016WS; left) and direct seeded (2018WS; right) traits on the Thai/SE Asia panel characterized in the study. Arrows indicate the values for the check variety KDML 105 in each treatment. WS: wet season, S: drought stress treatment, WW: well-watered treatment, H^2^: broad-sense heritability


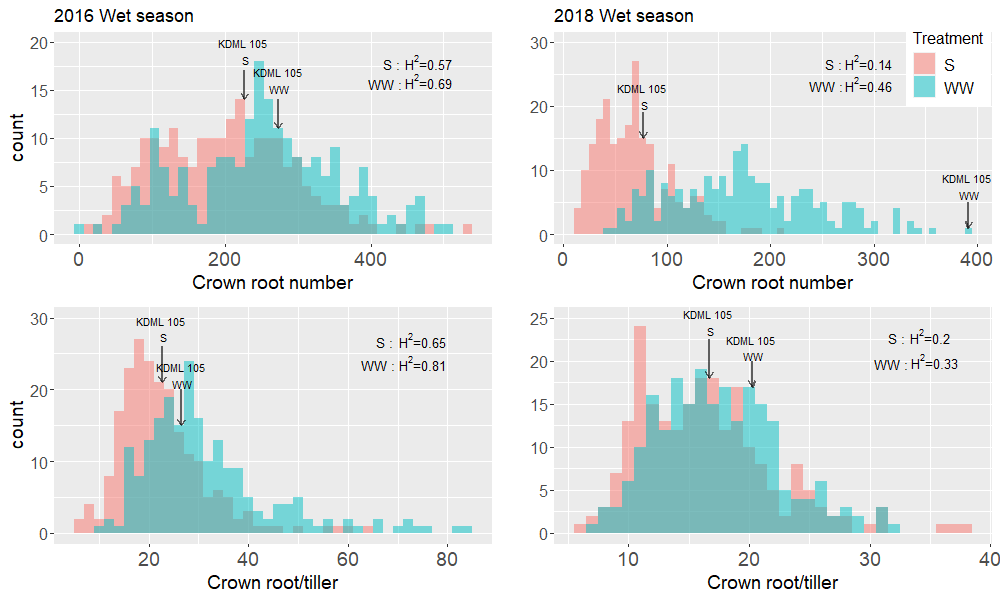


**Figure S3.** Distribution of crown root traits in the puddled, transplanted (2016WS; left) and direct seeded (2018WS; right) traits on the Thai/SE Asia panel characterized in the study. Arrows indicate the values for the check variety KDML 105 in each treatment. WS: wet season, S: drought stress treatment, WW: well-watered treatment, H^2^: broad-sense heritability


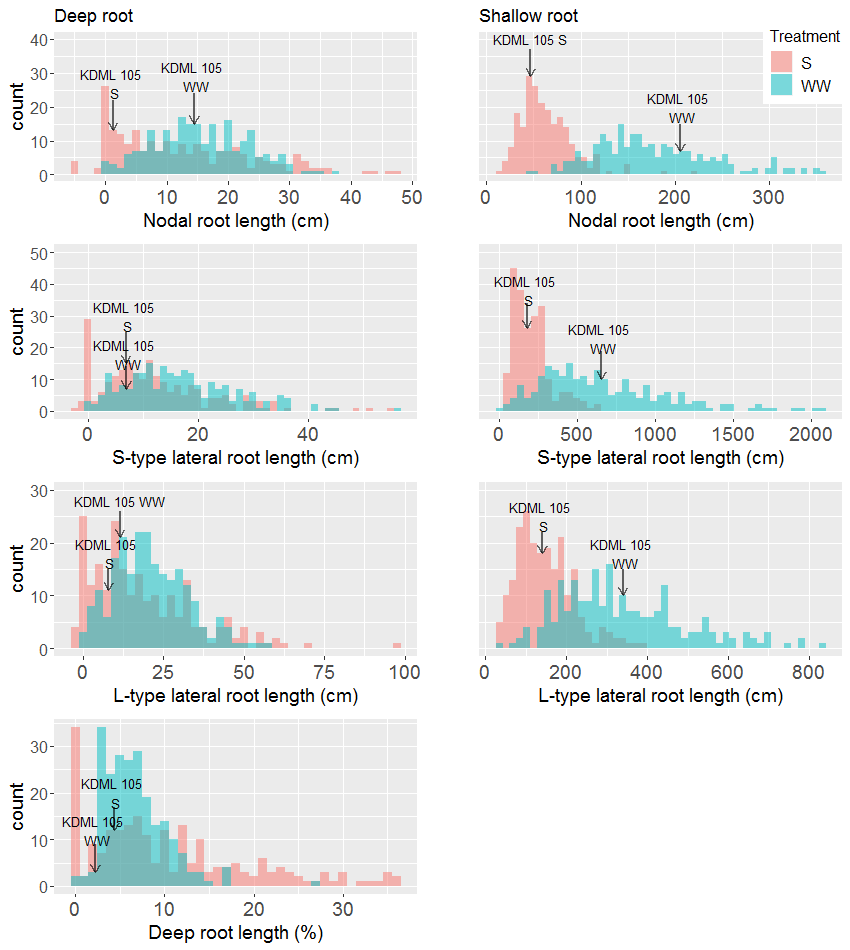


**Figure S4.** Distribution of root architectural traits from soil cores in the direct seeded trials (2018WS) on the Thai/SE Asia panel characterized in this study. Arrows indicate the values for the check variety KDML 105 in each treatment. WS: wet season, S: drought stress treatment, WW: well-watered treatment


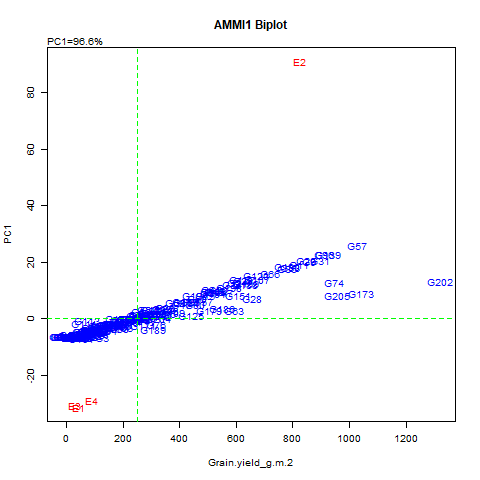


**Figure S5.** Biplot of AMMI analysis results on grain yield across 4 trials. PC1 values closer to 0 represent more stable grain yield, and larger lsmean values represent higher mean grain yield.

**
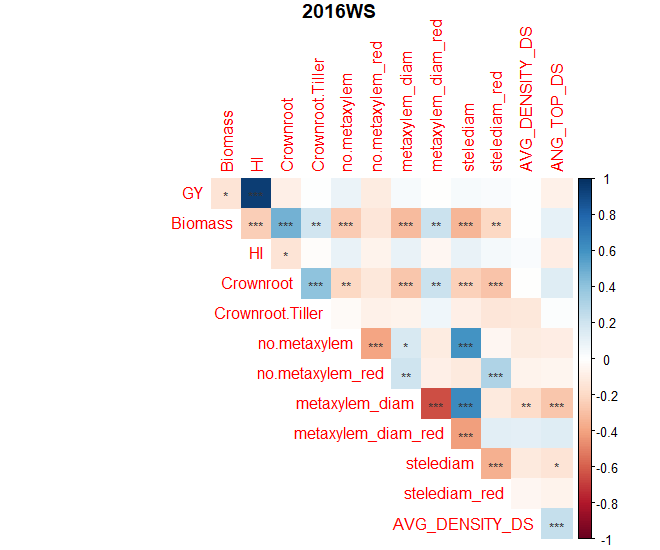

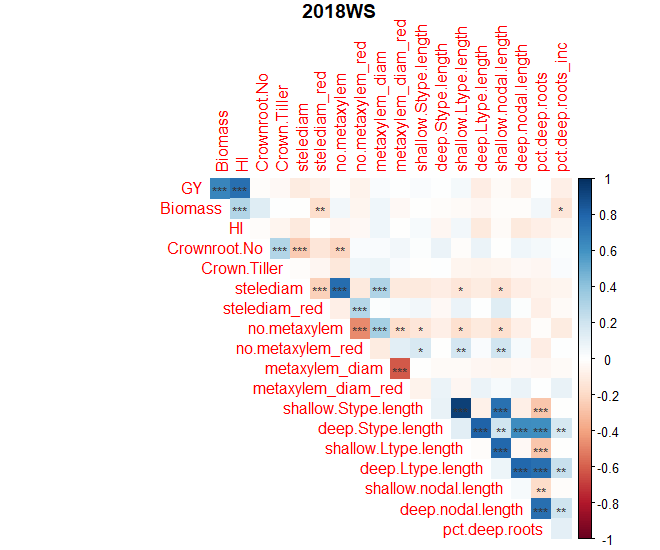
**

**Figure S6.** Correlation of traits in agronomic, root anatomical and morphological traits in puddled, transplanted (2016WS; left) and direct seeded (2018WS; right) trials on the Thai/SE Asia panel. All traits shown are from the drought stress treatment, and “red” indicates the relative reduction in the trait under drought stress as compared to the well-watered treatment. WS: wet season


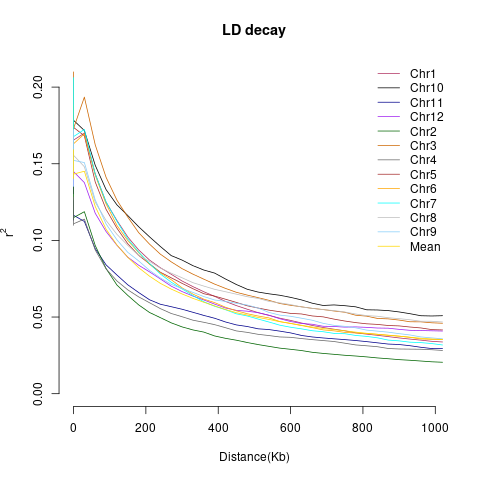


**Figure S7.** Linkage disequilibrium decay in Thai/SE Asia rice panel.


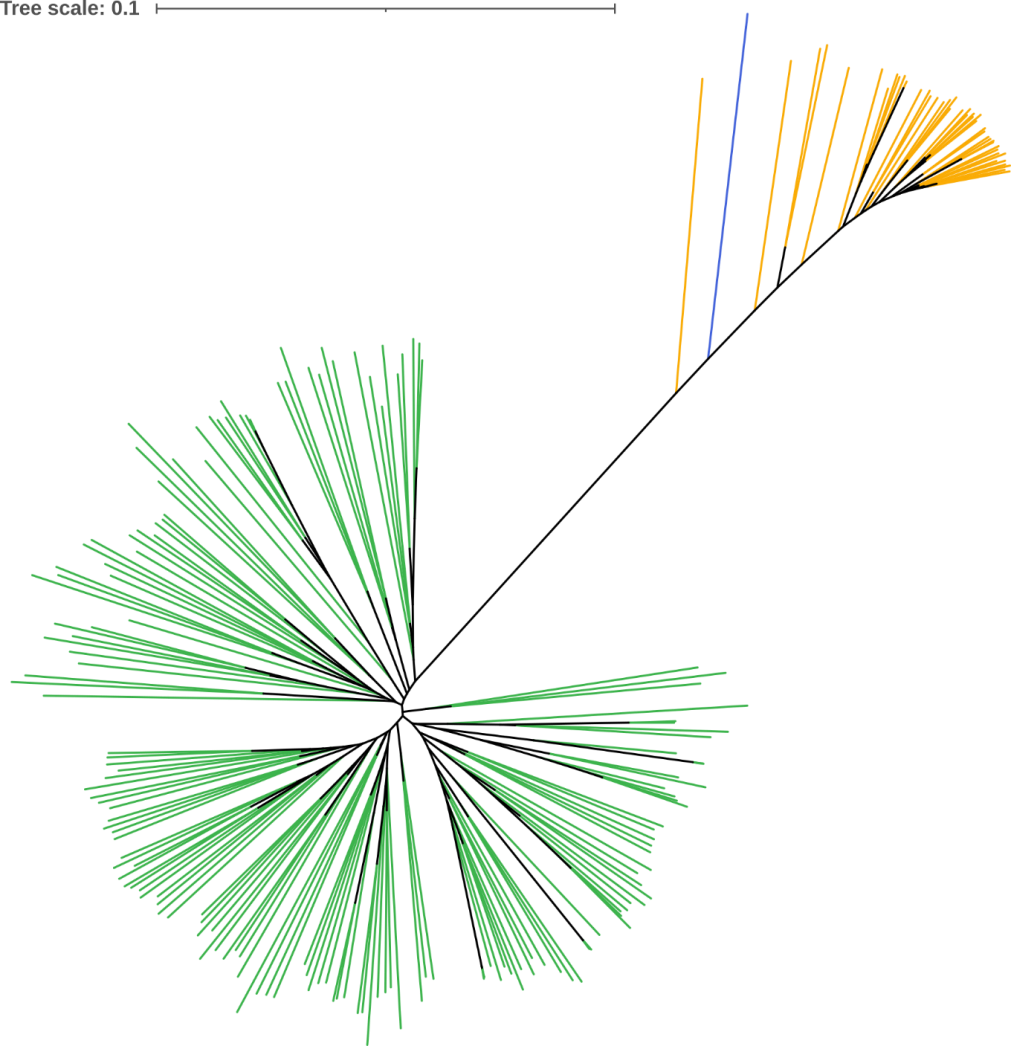


**Figure S8.** Phylogenetic tree of 209 Thai/SE Asia panel. Genotypes in green in the indica group, orange are in the japonica group, and the blue genotype is in the aromatic group.

**Figure S9.** GWAS results for root architectural traits in direct seeded (2018WS) trials on the Thai/SE Asia panel. WS: wet season

**Figure S10**. Performance of rice accessions carrying SNP 17349241 (T/C), 17349289 (C/T), 17349635 (G/A) and 17349299 (G/T) at gene Os06g0496400 (*OsRSL3*) and SNP 7407112 (C/T) at gene Os07g0232900 (*OsHMA3*) controlling median metaxylem diameter and their association with other phenotypes. **** denotes P<0.0001; ** denotes P<0.01; * denotes P<0.05; NS: not significant based on t-test with Bonferroni correction.
